# Multi-trait genome-wide association study identifies new loci associated with optic disc parameters

**DOI:** 10.1038/s42003-019-0634-9

**Published:** 2019-11-27

**Authors:** Pieter W. M. Bonnemaijer, Elisabeth M. van Leeuwen, Adriana I. Iglesias, Puya Gharahkhani, Veronique Vitart, Anthony P. Khawaja, Mark Simcoe, René Höhn, Angela J. Cree, Rob P. Igo, International Glaucoma Genetics Consortium, Kathryn P. Burdon, Jamie E. Craig, Alex W. Hewitt, Jost Jonas, Chiea-Cheun Khor, Francesca Pasutto, David A. Mackey, Paul Mitchell, Aniket Mishra, Calvin Pang, Louis R Pasquale, Henriette Springelkamp, Gudmar Thorleifsson, Unnur Thorsteinsdottir, Ananth C. Viswanathan, Robert Wojciechowski, Tien Wong, Terrri L Young, Tanja Zeller, NEIGHBORHOOD consortium, Rand Allingham, Don Budenz, Jessica Cooke Bailey, John Fingert, Douglas Gaasterland, Teresa Gaasterland, Jonathan L. Haines, Lisa Hark, Michael Hauser, Jae Hee Kang, Peter Kraft, Richard Lee, Paul Lichter, Yutao Liu, Syoko Moroi, Louis R. Pasquale, Margaret Pericak, Anthony Realini, Doug Rhee, Julia R. Richards, Robert Ritch, William K. Scott, Kuldev Singh, Arthur Sit, Douglas Vollrath, Robert Weinreb, Gadi Wollstein, Don Zack Wilmer, UK Biobank Eye and Vision Consortium, Denize Atan, Tariq Aslam, Sarah A. Barman, Jenny H. Barrett, Paul Bishop, Peter Blows, Catey Bunce, Roxana O. Carare, Usha Chakravarthy, Michelle Chan, Sharon Y. L. Chua, David P. Crabb, Philippa M. Cumberland, Alexander Day, Parul Desai, Bal Dhillon, Andrew D. Dick, Cathy Egan, Sarah Ennis, Paul Foster, Marcus Fruttiger, John E. J. Gallacher, David F. Garway, Jane Gibson, Dan Gore, Jeremy A. Guggenheim, Alison Hardcastle, Simon P. Harding, Ruth E. Hogg, Pearse A. Keane, Peng T. Khaw, Gerassimos Lascaratos, Tom Macgillivray, Sarah Mackie, Keith Martin, Michelle McGaughey, Bernadette McGuinness, Gareth J. McKay, Martin McKibbin, Danny Mitry, Tony Moore, James E. Morgan, Zaynah A. Muthy, Eoin O’Sullivan, Chris G. Owen, Praveen Patel, Euan Paterson, Tunde Peto, Axel Petzold, Jugnoo S. Rahi, Alicja R. Rudnikca, Jay Self, Sobha Sivaprasad, David Steel, Irene Stratton, Nicholas Strouthidis, Cathie Sudlow, Dhanes Thomas, Emanuele Trucco, Adnan Tufail, Stephen A. Vernon, Ananth C. Viswanathan, Cathy Williams, Katie Williams, Jayne V. Woodside, Max M. Yates, Jennifer Yip, Yalin Zheng, Aslihan Gerhold-Ay, Stefan Nickels, James F. Wilson, Caroline Hayward, Thibaud S. Boutin, Ozren Polašek, Tin Aung, Chiea Chuen Khor, Najaf Amin, Andrew J. Lotery, Janey L. Wiggs, Ching-Yu Cheng, Pirro G. Hysi, Christopher J. Hammond, Alberta A. H. J. Thiadens, Stuart MacGregor, Caroline C. W. Klaver, Cornelia M. van Duijn

**Affiliations:** 1 Department of Ophthalmology, Erasmus MC, Rotterdam, The Netherlands; 2 Department of Epidemiology, Erasmus MC, Rotterdam, The Netherlands; 3 The Rotterdam Eye Hospital, Rotterdam, The Netherlands; 4 Department of Clinical Genetics, Erasmus MC, Rotterdam, The Netherlands; 5 Statistical Genetics, QIMR Berghofer Medical Research Institute, Royal Brisbane Hospital, Brisbane, Australia; 6 Medical Research Council Human Genetics Unit, Institute of Genetics and Molecular Medicine, University of Edinburgh, Edinburgh, UK; 7 NIHR Biomedical Research Centre, Moorfields Eye Hospital NHS Foundation Trust and UCL Institute of Ophthalmology, London, UK; 8 Twin Research and Genetic Epidemiology, King’s College London, London, UK; 9 Department of Ophthalmology, Inselspital, University Hospital Bern, University of Bern, Bern, Germany; 10 Department of Ophthalmology, University Medical Center Mainz, Mainz, Germany; 11 Clinical and Experimental Sciences, Faculty of Medicine, University of Southampton, Southampton, UK; 12 Department of Ophthalmology, Harvard Medical School, Boston, MA USA; 13 Institute of Medical Biostatistics, Epidemiology and Informatics, University Medical Center Mainz, Mainz, Germany; 14 Centre for Global Health Research, The Usher Institute for Population Health Sciences and Informatics, University of Edinburgh, Edinburgh, UK; 15 Faculty of Medicine, University of Split, Split, Croatia; 16 Singapore Eye Research Institute, Singapore National Eye Centre, Singapore, Singapore; 17 Ophthalmology & Visual Sciences Academic Clinical Program, Duke-NUS Medical School, Singapore, Singapore; 18 Department of Ophthalmology, Yong Loo Lin School of Medicine, National University of Singapore, Singapore, Singapore; 19 Division of Human Genetics, Genome Institute of Singapore, Singapore, Singapore; 20 Department of Ophthalmology, Radboud Medical Center, Nijmegen, The Netherlands; 21 Institute for Molecular and Clinical Ophthalmology, Basel, Switzerland; 22 Nuffield Department of Public Health, University of Oxford, Oxford, UK; 23 Menzies Institute for Medical Research, University of Tasmania, Hobart, Tasmania Australia; 24 Department of Ophthalmology, Flinders University, Adelaide, Australia; 25 Centre for Eye Research Australia, University of Melbourne, Royal Victorian Eye and Ear Hospital, Melbourne, Australia; 26 Department of Ophthalmology, Medical Faculty Mannheim of the Ruprecht-Karls-University of Heidelberg, Mannheim, Germany; 27 Institute of Human Genetics, Friedrich-Alexander-Universität Erlangen-Nürnberg (FAU), Erlangen, Germany; 28 Centre for Ophthalmology and Visual Science, Lions Eye Institute, University of Western Australia, Perth, Australia; 29 Centre for Vision Research, Department of Ophthalmology and Westmead Millennium Institute, University of Sydney, Sydney, Australia; 30 Statistical Genetics, Queensland Institute of Medical Research, Brisbane, 4029 Australia; 31 Department of Ophthalmology and Visual Sciences, Chinese University of Hong Kong, Hong Kong, China; 32 Department of Ophthalmology, Mt. Sinai School of Medicine, New York, NY USA; 33 deCODE Genetics/Amgen, 101 Reykjavik, Iceland; 34 Department of Epidemiology, Johns Hopkins Bloomberg School of Public Health, Baltimore, MD USA; 35 Wilmer Eye Institute, Johns Hopkins Bloomberg School of Public Health, Baltimore, MD USA; 36 National Human Genome Research Institute (NIH), Baltimore, MD USA; 37 Department of Ophthalmology, Singapore National Eye Centre, Singapore, Singapore; 38 Department of Ophthalmology and Visual Sciences, School of Medicine and Public Health, University of Wisconsin, Madison, WI USA; 39 Clinic for General and Interventional Cardiology, University Heart Center Hamburg, Hamburg, Germany; 40 University of Bristol, Bristol, UK; 41 Manchester University, Manchester, UK; 42 Kingston University, Kingston, UK; 43 University of Leeds, Leeds, UK; 44 NIHR Biomedical Research Centre, London, UK; 45 King’s College London, london, UK; 46 University of Southampton, Southampton, UK; 47 Queens University Belfast, Belfast, UK; 48 University College London, London, UK; 49 University College London Great Ormond Street Institute of Child Health, London, UK; 50 University of Edinburgh, Edinburgh, UK; 51 University of Oxford, Oxford, UK; 52 Cardiff University, Cardiff, UK; 53 University of Liverpool, Liverpool, UK; 54 University of Cambridge, Cambridge, UK; 55 Leeds Teaching Hospitals NHS Trust, Leeds, UK; 56 King’s College Hospital NHS Foundation Trust, London, UK; 57 University of London, London, UK; 58 Newcastle University, Newcastle, UK; 59 Gloucestershire Hospitals NHS Foundation Trust, Gloucester, UK; 60 University of Dundee, Dundee, UK; 61 Nottingham University Hospitals NHS Trust, Nottingham, UK; 62 University of East Anglia, Norwich, UK; 63 Department of Ophthalmology, Duke University Medical Center, Durham, NC USA; 64 Murray Brilliant Center for Human Genetics, Marshfield Clinic Research Foundation, Marshfield, WI USA; 65 Department of Ophthalmology, University of North Carolina, Chapel Hill, NC USA; 66 Department of Epidemiology and Biostatistics, Institute for Computational Biology, Case Western Reserve University School of Medicine, Cleveland, OH, USA; 67 Department of Ophthalmology, University of Iowa, College of Medicine, IA, Iowa USA; 68 Department of Anatomy and Cell Biology, University of Iowa, College of Medicine, IA, Iowa USA; 69 Eye Doctors of Washington, Chevy Chase, MD, USA; 70 Scripps Genome Center, University of California at San Diego, San Diego, CA USA; 71 Department of Ophthalmology, Sidney Kimmel Medical College, Philadelphia, PA USA; 72 Department of Medicine, Duke University Medical Center, Durham, NC USA; 73 Channing Division of Network Medicine, Brigham and Women’s Hospital, Harvard Medical School, Boston, MA USA; 74 Department of Epidemiology, Harvard School of Public Health, Boston, MA USA; 75 Program in Genetic Epidemiology and Statistical Genetics, Harvard School of Public Health, Boston, MA USA; 76 Bascom Palmer Eye Institute, University of Miami Miller School of Medicine, Miami, FL USA; 77 Department of Ophthalmology and Visual Sciences, University of Michigan, Ann Arbor, MI USA; 78 Department of Cellular Biology and Anatomy, Georgia Regents University, Augusta, GA USA; 79 James and Jean Culver Vision Discovery Institute, Georgia Regents University, Augusta, GA USA; 80 Vance Institute for Human Genomics, University of Miami Miller School of Medicine, Miami, FL USA; 81 Department of Ophthalmology, West Virginia University Eye Institute, Morgantown, WV USA; 82 Department of Ophthalmology, Case Western Reserve University School of Medicine, Cleveland, OH, USA; 83 Department of Epidemiology, University of Michigan, Ann Arbor, MI USA; 84 Einhorn Clinical Research Center, Department of Ophthalmology, New York Eye and Ear Infirmary of Mount Sinai, New York, NY USA; 85 Joel Schuman Department of Ophthalmology, NYU School of Medicine, New York, NY USA; 86 Institute for Human Genomics, University of Miami Miller School of Medicine, Miami, FL USA; 87 Department of Ophthalmology, Stanford University School of Medicine, Palo Alto, CA USA; 88 Department of Ophthalmology, Mayo Clinic, Rochester, MN USA; 89 Department of Genetics, Stanford University School of Medicine, Palo Alto, CA USA; 90 Department of Ophthalmology, University of California San Diego, San Diego, CA USA; 91 Department of Ophthalmology, NYU School of Medicine, New York, NY USA; 92 Eye Institute, Johns Hopkins University Hospital, Baltimore, MD USA

**Keywords:** Genome-wide association studies, Optic nerve diseases

## Abstract

A new avenue of mining published genome-wide association studies includes the joint analysis of related traits. The power of this approach depends on the genetic correlation of traits, which reflects the number of pleiotropic loci, i.e. genetic loci influencing multiple traits. Here, we applied new meta-analyses of optic nerve head (ONH) related traits implicated in primary open-angle glaucoma (POAG); intraocular pressure and central corneal thickness using Haplotype reference consortium imputations. We performed a multi-trait analysis of ONH parameters cup area, disc area and vertical cup-disc ratio. We uncover new variants; rs11158547 in *PPP1R36-PLEKHG3* and rs1028727 near *SERPINE3* at genome-wide significance that replicate in independent Asian cohorts imputed to 1000 Genomes. At this point, validation of these variants in POAG cohorts is hampered by the high degree of heterogeneity. Our results show that multi-trait analysis is a valid approach to identify novel pleiotropic variants for ONH.

## Abstract (web summary)

The International Glaucoma Genetics Consortium reports a genome-wide association study meta-analysis of optic disc parameters relevant to primary open-angle glaucoma. They identify two novel variants in *PPP1R36-PLEKHG3* and *SERPINE3* by multi-trait analysis in European-ancestry cohorts that were replicated in independent Asian cohorts.

## Introduction

Glaucoma is the most common cause of irreversible blindness in the world^1^. Primary open angle glaucoma (POAG) is the most prevalent type of glaucoma accounting for 74% of all glaucoma cases^2,3^. Intraocular pressure (IOP) and the morphology of the optic nerve head (cup area (CA), disc area (DA), and vertical cup–disc ratio (VCDR)) are important features of the glaucomatous process. For each of these traits, twin studies showed a high heritability (*h*^2^_CA_ = 0.75, *h*^2^_DA_ = 0.72, *h*^2^_IOP_ = 0.55, and *h*^2^_VCDR_ = 0.48)^4^. Central corneal thickness (CCT) is also a highly heritable trait (*h*^2^_CCT_ = 0.68–0.95)^5^, which is most likely non-physiologically associated with POAG, but rather biases IOP measurement, the major risk factor of POAG^6,7^. CA, DA, and VCDR, are significantly correlated both at the genetic level^8^. The high genetic correlation found between the optic nerve head (ONH) traits (*R*_g_ = 0.31–0.83) raises the question whether multi-trait analyses will improve the statistical power of the individual GWAS and will find variants with pleiotropic effects^9^.

For this study, we generated new data on these 5 quantitative traits by imputing 12 European ancestry cohorts from the International Glaucoma Genetic Consortium (IGGC) (*n*_MAX_ = 31,269) to haplotype reference consortium (HRC) release 1 imputation panel, which includes over 39 million variants^10^. A meta-analysis of these 12 European ancestry studies served as a discovery cohort in the analyses. Replication was performed in five Asian ancestry cohorts that were part of the IGGC. The cohorts of Asian descent were imputed to 1000 Genomes as there is little gain in HRC imputation in this ancestry group because there are no additional Asian samples included in HRC (http://www.haplotype-reference-consortium.org/participating-cohorts)^11^. We evaluated the added value of multi-trait analyses using two programs: CPASSOC and multi-trait analysis of GWAS (MTAG). Both use aggregated GWAS results. Whereas CPASSOS performs a meta-analysis assuming homogeneous and heterogeneous effects across traits by applying a inter-trait correlation matrix, MTAG basically increases the power of a single trait analyses by incorporating the GWAS findings of correlated traits.

By multi-trait analysis we identified two novel loci associated with the ONH at rs11158547 in-between *PPP1R36* and *PLEKHG3* and at rs1028727 near *SERPINE3* in those of European descent. These loci replicated in the Asian replication sample. Findings for these loci were consistent using a distinct multi-trait approach, MTAG, in both the European and Asian cohorts. This study emphasis that multi-trait analysis in GWAS pleiotropic traits is an effective approach to identify variants harboring correlated traits

## Results

### Replication of previous CA, DA, VCDR, IOP, and CCT GWAS results

As a validation we first confirmed previously identified loci for CA, DA, VCDR, IOP, and CCT by Springelkamp et al.^8^ (*n*_CA_ = 22,489; *n*_DA_ = 22,504; *n*_VCDR_ = 23,899; *n*_IOP_ = 37,930) and Iglesias et al.^12^ (*n* = 17,803) based on 1000 Genomes imputation. Supplementary Fig. 1 and Supplementary Datas 1 and 2 show the per trait comparison of our meta-analysis of all European ancestry discovery cohorts using the HRC imputation with the results of the meta-analysis by Springelkamp^8^ and Iglesias^12^ based on 1000 Genomes. Out of 113 (95%), 107 available variants in HRC replicated at a Bonferroni significance level.

### Optic nerve head parameters

**Fig. 1:**
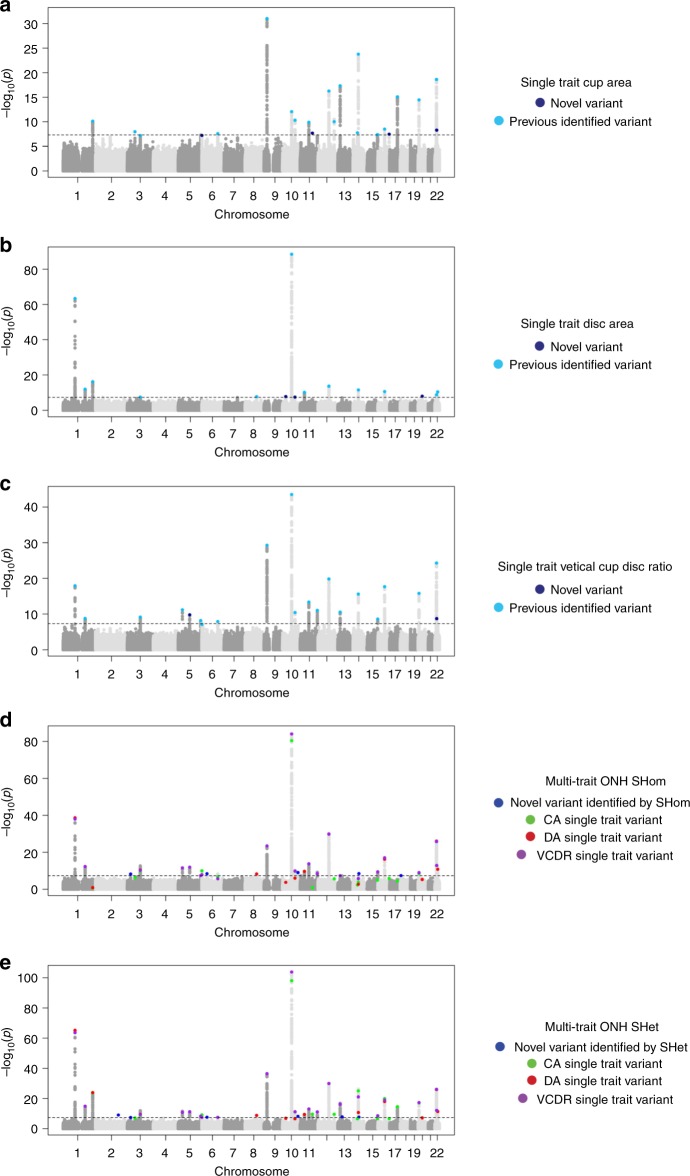
Manhattan plot of single trait analysis for cup area (**a**), disc area (**b**), and vertical cup–disc ratio (**c**). Manhattan plot for multi-trait analysis of the optic nerve head (ONH) SHom (**d**) and SHet (**e**).

**Fig. 2:**
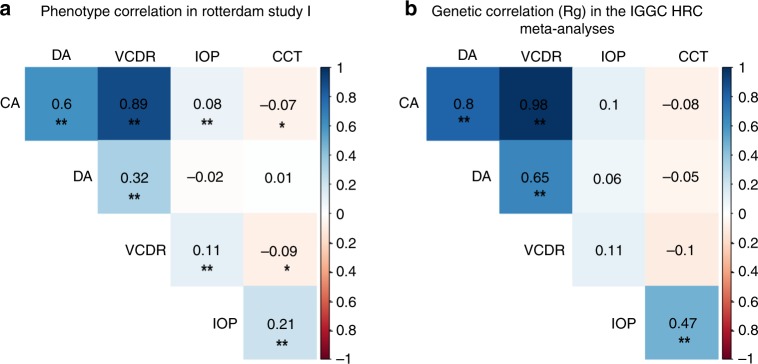
Phenotype (**a**) and genetic (**b**) correlations between cup area, disc area, vertical cup–disc ratio, intraocular pressure, and central corneal thickness. **a** Partial pearson correlation coefficient s between cup area (CA), disc area (DA), vertical cup–disc ratio (VCDR), intraocular pressure (IOP), and central corneal thickness (CCT) adjusted for age and sex in the Rotterdam study I. **b** Genetic correlation coefficient (Rg) for CA, DA, VCDR, IOP, and CCT calculated by LD score regression; *p < 0.05, **p < 0.0001.

In the single trait meta-analyses of ONH traits (CA, DA, and VCDR) in those of European descent (*n*_CA_ = 24,493, LDSC intercept_ca_ = 1.024 (SE = 0.0083); *n*_DA_ = 24,509, LDSC intercept_DA_ = 1.041 (SE = 0.0071); *n*_VCDR_ = 25,180, LDSC intercept_VCDR_ = 1.029 (SE = 0.0081) Supplementary Data 3), 59 loci showed genome-wide significant association with at least one of the traits (Fig. 1a–c, Supplementary Data 4). The ONH analyses yielded six loci not previously reported (Table 1, Supplementary Data 5), however, none of these novel variants replicated in the Asian replication sample comprising five Asian studies. As the correlation analysis between the ONH traits showed significant correlations at the genetic and phenotype level (Fig. 2), we applied multi-trait analysis to uncover pleiotropic effects. Using multi-trait approach CPASSOC^13^, we identified three new loci at *p* < 5 × 10^−8^ by CPASSOC’s SHom (*KIF6*, *EPB41L3*, *PPP1R36-PLEKHG3*) (Fig. 1d, Supplementary Data 6). This method assumes that genetic effects are homogenous across traits and cohorts. Two additional new loci were identified by SHet (*ZAK*, *SERPINE3*) (Fig. 1e, Supplementary Data 6), which assumes the genetic effects are heterogenous. Locuszoom plots for these novel variants a depicted in Supplementary Fig. 2. Using an alternative approach (MTAG)^14^, the loci emerged consistently as genome-wide significant: rs9471130 near *KIF6* in the DA analysis (*p* = 2.63 × 10^−^^08^), rs11158547 near *PPP1R36-PLEKHG3* in the CA analysis (*p* = 2.13 × 10^−08^) and rs1028727 near *SERPINE3* in the DA analysis (*p* = 4.50 × 10^−09^) (Supplementary Data 7). rs11158547 (*PPP1R36-PLEKHG3*) and rs1028727 (*SERPINE3*) displayed nominally significant association in the multi-trait analysis (CPASSOC and MTAG) in individuals of Asian ancestry (Supplementary Datas 6 and 7). Both variants were not in LD (*r*^2^ < 0.1) with neighboring known variants near *SIX6*, *DDHD1*, and *DLCK1*.

**Table 1:** Genome-wide significant SNPs newly identified for cup area, disc area, vertical cup–disc ratio, intraocular pressure or central corneal thickness in the European HRC discovery.

Trait	rsID	Chr:pos	Nearest Gene	EA	*N*_j_	Freq	*β*	SE	*p* Value	*I*^2^	HetP	*β*_j_	SE_j_	*p* Value_j_
DA	rs4748969	10:25015618	*ARHGAP21*	A	25126	0.277	−0.025	0.004	1.69E−08	0	0.568	−0.025	0.004	1.77E−08
DA	rs10882283	10:95360964	*RBP4*	C	23525	0.377	−0.023	0.004	3.68E−08	0	0.864	−0.023	0.004	3.38E−08
CA	rs7101609	11:92623493	*FAT3*	G	26056	0.354	−0.013	0.002	2.06E−08	0	0.772	−0.013	0.002	1.25E−08
CA	rs1622797	16:86379107	*LINC00917*	T	24593	0.089	0.022	0.004	3.41E−08	45	0.069	0.022	0.004	3.87E−08
DA	rs6119893	20:31142813	*C20orf112*	T	25347	0.327	−0.024	0.004	1.06E−08	0	0.542	−0.024	0.004	9.78E−09
CA^a^	rs2412973	22:30529631	*HORMAD2*	A	26675	0.442	0.013	0.002	5.06E−09	45.9	0.063	0.013	0.002	3.89E−09
VCDR^a^	rs2412973	22:30529631	*HORMAD2*	A	27448	0.442	0.008	0.001	1.97E−09	70.2	0.001	0.008	0.001	1.96E−09
VCDR	rs115456027	5:87919700	*LINC00461*	T	25273	0.078	0.016	0.003	1.66E−10	49.2	0.046	0.016	0.003	2.08E−10
CA^b^	rs17135931	6:625188	*EXOC2*	A	26267	0.189	0.015	0.003	6.25E−08	0	0.537	0.015	0.003	3.86E−08
IOP	rs9853115	3:186131600	*RP11-78H24.1*	A	32544	0.496	−0.158	0.027	2.85E−09	0	0.666	−0.158	0.027	2.91E−09
IOP	rs150202082	18:53027723	*TCF4*	T	30915	0.027	−0.47	0.085	2.97E−08	17.9	0.273	−0.47	0.085	3.06E−08
CCT	rs34869	5:115152694	*CDO1*	C	17810	0.437	2.797	0.397	1.97E−12	0	0.93	2.797	0.398	2.1E−12
CCT	rs1772570	13:81193433	*HNRNPA1P31*	C	18158	0.315	−2.368	0.42	1.74E−08	37.4	0.101	−2.368	0.421	1.79E−08
CCT	rs511651	18:24357736	*AQP4-AS1*	C	18457	0.319	2.452	0.416	3.81E−09	0	0.644	2.452	0.416	3.93E−09

*DA* disc area, *CA* cup area, *VCDR* vertical cup–disc ratio, *IOP* intraocular pressure, *CCT* central corneal thickness. The position (Chr:pos) of the variant is the position in GRCh37/hg19. The Freq column is the frequency of the effect allele (EA) and the *β* column is the effect of the effect allele. *N* is the effective sample size and is determined by GCTA. *β*_j_, SEβ_j_, and *p* value_j_ are the effect size, standard error, and *p* value from a joint analysis of all the selected SNPs, as determined by GCTA. The per cohort statistics can be found in the Supplementary Data 5.

^a^Variant previously identified for DA by Springelkamp et al.^38^

^b^Variant previously identified for VCDR by Springelkamp et al.^39^

### IOP and CCT

Next, we conducted a single trait meta-analysis for IOP and for CCT, the two traits that are not likely physiologically related. For IOP, we meta-analyzed a total of 31,269 participants (LDSC intercept = 1.028; SE = 0.0078, Supplementary Data 4) and identified 9 genome-wide significant regions of which two were novel in the HRC-based imputations and had not been uncovered in the IGGC 1000 Genomes analyses before (Table 1, Supplementary Data 5). The lead single-nucleotide polymorphisms (SNPs) in these genomic regions were a common variant rs9853115 near *DGKG* on 3q27.3 and a rare variant rs150202082[T] (frequency 0.03) near *TCF4* on 18q21.2. rs9853115 failed replication in the Asians (*p* = 0.9315) and rs150202082 could not be examined since this variant was monoallelic in the Asian individuals. A GWAS by Choquet et al.^15^ also identified rs9853115 as new variant associated with IOP in multiethnic cohort of predominately (83%) European ancestry. The same study also identified a novel variant near *TCF4*, rs11659764, approximately 300 kb upstream of rs150202082 which was in relatively weak LD (*r*^2^ = 0.4). In a recent study from the UKbiobank by Khawaja et al.^16^ the same variant near *DGKG* showed genome-wide significant association with similar effectsize, however, rs150202082 near *TCF4* could not be validated in this study.

In the meta-analysis of CCT, a total of 16,204 participants were included (LDSC intercept = 0.989; SE = 0.0082). We identified 31 independent genome-wide significant signals of which three were novel (Table 1 and Supplementary Data 5), including a variant, rs34869, near *CDO1*. Again, none of the three new variants replicated at a nominal significance level in the Asian samples. Multi-trait analysis by CPASSOC identified four novel variants (Supplementary Datas 8 and 9). In contrast to ONH cross trait analyses, also these could not be replicated in the Asian.

### In silico analysis

**Fig. 3:**
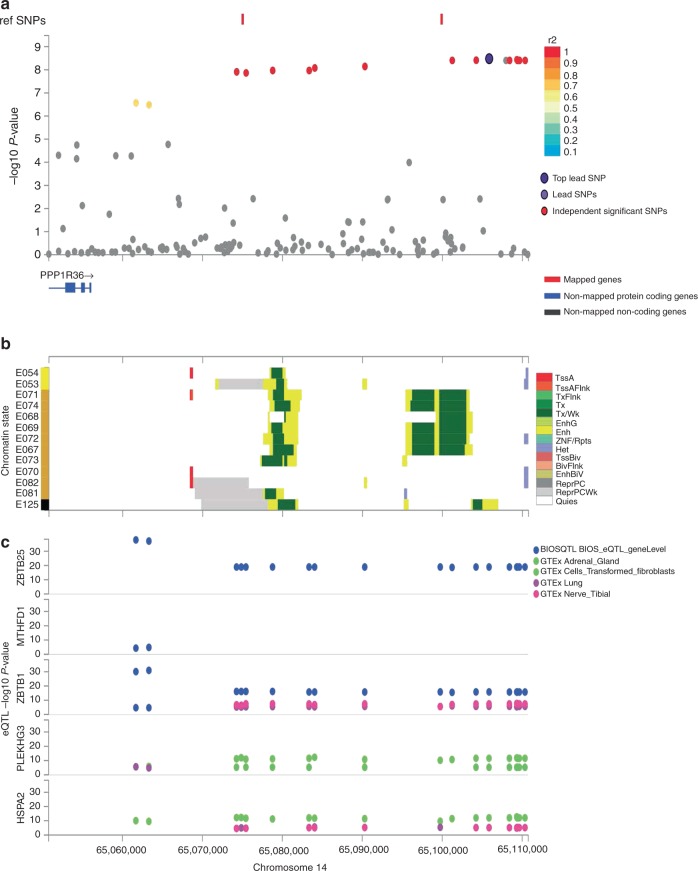
Regional, chromatin state, and eQTL plot for rs11158547 (*PPP1R36-PLEKHG3*). Panel **a** shows the regional assocaiations plots with −log10 *p* value depicted on the *y*-axis, genes mapped by either position, eQTL or chromatin interaction are depicted in red on the *x*-axis; panel **b** shows 15 core chromatin states of varaints plotted in panel **a** for 13 brain tissues from Roadmap epidenomes described on the *y*-axis. E054 ganglion eminence derived primary cultured neurospheres, E053 cortex-derived primary cultured neurospheres, E071 brain hippocampus middle, E074 brain substantia nigra, E068 brain anterior caudate, E069 brain cingulate gyrus, E072 brain inferior temporal lobe, E067 brain angular gyrus, E073 brain dorsolateral prefrontal cortex, E070 brain germinal matrix, E082 fetal brain female, E081 fetal brain male, E125 NH-A astrocytes primary cells. Panel **c** depicts varaints that overlap eQTLs from selected eQTL databases described in the legend of panel (**c**).

**Fig. 4:**
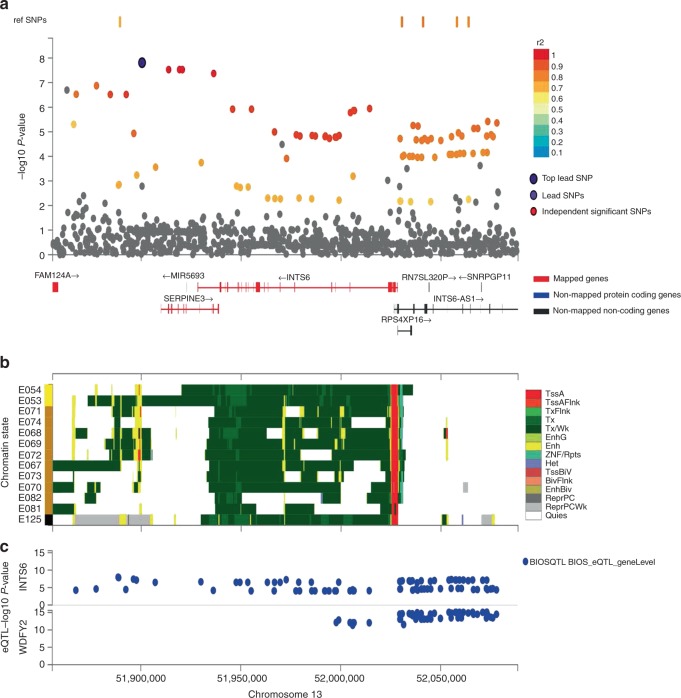
Regional, chromatin state and eQTL plot for rs1028727 (*SERPINE3*). Panel **a** shows the regional assocaiations plots with −log10 *p* value depicted on the *y*-axis, genes mapped by either position, eQTL or chromatin interaction are depicted in red on the *x*-axis; panel **b** shows 15 core chromatin states of varaints plotted in panel **a** for 13 brain tissues from Roadmap epidenomes described on the *y*-axis. E054 ganglion eminence derived primary cultured neurospheres, E053 cortex-derived primary cultured neurospheres, E071 brain hippocampus middle, E074 brain substantia nigra, E068 brain anterior caudate, E069 brain cingulate gyrus, E072 brain inferior temporal lobe, E067 brain angular gyrus, E073 brain dorsolateral prefrontal cortex, E070 brain germinal matrix, E082 fetal brain female, E081 fetal brain male, E125 NH-A astrocytes primary cells. Panel **c** depicts varaints that overlap eQTLs from selected eQTL databases described in the legend of panel (**c**).

To investigate the functional and regulatory potential, we annotated the variants in linkage disequilibrium (European LD, *r*^2^ ≥ 0.8) with the lead SNPs at the two new and replicated ONH variants, rs11158547 and rs1028727, using a combination of bioinformatics tools (see Method section). A total of 70 variants in LD with the 2 novel variants were queried. None of the examined variants were predicted to damage protein structure by SIFT, Polyphen, or alternative splicing using Ensembl’s Variant Effect Predictor. As all queried variants are noncoding, we reviewed the possible regulatory annotation of these SNPs in experimental epigenetic evidence, including DNAse hypersensitive sites, histone modifications, and transcription factor-binding sites in human cell lines and tissues from the ENCODE^17^ and ROADMAP EPIGENOMICS^18^ projects, integrated in Haploreg^19^. Annotations of chromatin states indicated that the two novel variants were located in, or in LD with, an active chromatin state region from at least one of the tissues investigated (Supplementary Data 10 and Figs. 3 and 4 for chromatin states in brain tissue). Next, we evaluated the overlaps of *cis*-expression quantitative trait loci (eQTL) in several databases (see Methods). In both novel loci *PPP1R36-PLEKHG3 and SERPINE3*, variants were found to be eQTL’s and based on Regulome DB-scores both ONH loci contained variants that were likely to alter binding (Supplementary Data 10).

### Gene prioritization, pathway analysis, and gene expression

We explored possible tissue expression and biological functions by pathway analysis for the two novel SNPs. We annotated these SNPs to genes by positional gene mapping, eQTL mapping and chromatin interaction mapping strategies implemented in FUMA^20^ (see Method section). For, rs11158547 (*PPP1R36-PLEKHG3*) 9 genes were assigned to this locus and for rs1028727 (*SERPINE3*) 21 genes were mapped to this locus (Supplementary Data 11). Pathway analysis based on enrichment of gene-set terms (MsigDB^21^ and Wikipathways^22^) found 5 and 11 Bonferroni significant gene-sets comprising genes mapped to *SERPINE3* locus and *PPP1R36-PLEKHG3* locus respectively. These pathways were in particular involved in immune response and cancer development (Supplementary Data 12 highlighted gene-sets).

As expression in eye tissues is not available in GTex^23^, we assessed the Ocular Tissue Database^24^. For 26 out of the 30 genes mapped to either rs11158547 or rs1028727 expression data were present in the Ocular Tissue Database (Supplementary Data 11). The highest levels of expression in the optic nerve was found for *HSPA2*, a gene associated to rs11158547 via lung, tibial nerve and fibroblasts eQTL’s and chromatin interaction mapping (Supplementary Fig. 3).

### From endophenotypes to glaucoma

We also investigated the translational potential of these two loci to POAG by carrying out a meta-analysis of three POAG studies, NEIGHBOR, Southampton and UK Biobank Eye and Vision Consortium (Ncase = 9450; Ncontrol = 436,824), from European origin. For rs1028727 a negative effect on the VCDR in the present study predicts a decreased risk of POAG which was seen in NEIGHBOR study and the UKBiobank but not in the Southampton study (Supplementary Data 13). rs11158547 in *PPP1R36-PLEKHG3* is predicted to be associated with increased POAG risk based on the positive effect of VCDR. Indeed in all three POAG studies the effect of the SNP is also positive. Pooling the studies based on a fixed effect analysis yields an OR 1.28 (95% CI: 1.15–1.39; *p* = 4.83 × 10^−8^) and showed Bonferroni significance (*p* = 0.025). Given the high degree of heterogeneity of effects at this locus a random effect meta-analysis was carried out which could not confirm this finding in POAG (Supplementary Data 14). Thus the findings were partly but not consistently replicated, awaiting larger and more homogeneous data sets for the final replication.

## Discussion

Our results implicate two novel loci, one downstream *SERPINE3* and one other in-between *PPP1R36* and *PLEKHG3*, both associated with ONH morphology via CPASSOC multi-trait analysis. *SERPINE3* belongs to the clade E family of extracellular serpins. Family members have been described to play a role in other neurodegenerative diseases such as Alzheimer’s disease^25^. Recent studies in glaucomatous human postmortem samples and in rat models identified oxidative inactivation of serpins (neuroserpin) as a molecular mechanism of increased plasmin activity leading to neurodegeneration in high ocular pressure conditions^26^. In the trabecular meshwork, serpins (plasminogen activator inhibitor) may mediate the inhibition of matrix metaloprotienase (MMPs) activity induced by transforming growth factor-beta enhancement^27,28^. Inactivity of MMPs were found to increase aqueous humor outflow resistance leading to rising IOP^29^. rs11158547 downstream the *PLEKHG3* gene, a pleckstrin homology domain containing protein, is also a relatively unknown gene. It contains a guanide nucleotide exchange factor (GEF) domain which is important for Rho-dependent signal transduction^30^. In mice *PLEKHG3* knockout is associated with an abnormal anterior chamber depth of the eye (IMPC release 3.2 http://www.mousephenotype.org/data/experiments?geneAccession=MGI:2388284). The other gene close to this locus, *PPP1R36*, is less likely a candidate gene for ONH. *PPP1R36*, has been described in connection with autophagy during spermatogenesis. *PPP1R36* encodes a regulatory subunit of protein phosphatase 1 which is involved in multiple cellular functions such as metabolism, immune response, apoptosis, meiosis, mitosis, cytoskeletal reorganization, and synthesis. However, its function to the eye has not been characterized.

A general insight from this study was that the strength of genetic correlation is an important condition for investigating the pleiotropic effect of genetic variants on traits. The high genetic and phenotypic correlation observed between the ONH traits enabled multi-trait analyses and yielded plausible and replicable results. We validated these novel variants identified by CPASSOC with another multi-trait analysis method MTAG, which also uses summary statistics as input. The main difference between both methods is that CPASSOC test whether a SNP is not associated with any of the traits under the null hypothesis. MTAG, on the contrary produces trait-specific effect estimates for each SNP. The variants discovered in the European CPASSOC analysis that replicated in the Asian CPASSOC analysis also replicated in the MTAG analysis. Sensitivity analysis excluding the Rotterdam studies showed a high correlation of the Shom and Shet statistic with the SHom/SHet statistic from the full analysis including the Rotterdam studies (*r* = 0.71, *p* < 2.2 × 10^−16^; *r* = 0.68, *p* < 2.2 × 10^−16^, respectively). The correlation in MTAG for CA, DA, and VCDR was considerably low yet very significant (*r*_CA_ = 0. 31, *p* < 2.2 × 10^−16^; *r*_DA_ = 0.56, *p* < 2.2 × 10^−16^, *r*_vcdr_ = 0.17).

In contrast, the multi-trait analysis between IOP and CCT could not uncover robust new variants. A reason for this observation might be the moderate magnitude of the genetic correlation between IOP and CCT. Also, clinical research has shown that this relation is largely driven by measurement errors in Goldmann applanation tonometry, rather than a pathophysiological process^31,32^.

A potential limitation of this study is the application of a different imputation panel for the discovery and replication phase. The European studies were all imputed to the HRC panel which has a beneficial imputation quality compared to 1000 Genomes. By contract the Asian replications set, was imputed to 1000 Genomes since a recent publication showed that HRC imputations perform less adequate in Asians^11^. Variants associated with cup area and other endophenotypes at genome-wide significance in the European single trait analysis could not be replicated in the Asian replication sample. The use of different imputation panels may be a source bias hampering replication. A theoretical shortcoming, is that the various studies used different methods and equipment to assess ONH parameters among studies. This has most likely reduced the power of the study and has generated most probably false negative rather than false-positive results. To prevent false-positive findings using novel methods, we aimed to replicate the findings of the primary CPASSOC analyses by another analyses using MTAG. The variants discovered in the CPASSOC analysis could also be replicated in MTAG. As both methods are mathematically distinct we concluded that our results are rather robust and independent of the statistical approach. This underscores the strength of the association as it is consistently found by two independent approaches.

We have no doubt that association of the variants to ONH are of interest to the biology community. To evaluate the implications of our findings in the context of glaucoma we studied three independent POAG studies. Up until now we are not able to link our finding to POAG. Although fixed effect meta-analysis showed Bonferroni significance (*p*  = 0.025) for rs11158547 in *PPP1R36-PLEKHG3*, random effect meta-analysis that takes into account the heterogeneity could not confirm this finding in POAG. The source of the high variability in estimates is unknown and may involve clinical variability and ethnic differences. It is important to realize that the identification of POAG genes is far from complete and work in progress.

In conclusion, we conducted single and multi-trait meta-analysis of five endophenotypes of glaucoma based on HRC imputations in European ancestral populations. The HRC single trait analyses in those of European descent did not yield new loci that could be replicated in Asians. We identified two novel loci for ONH in-between *PPP1R36-PLEKHG3* at chromosome 14q23.3 and near *SERPINE3* at chromosome 13q14.3 by multi-trait analysis in those of European descent that could be replicated in Asians using CPASSOC. Findings for these loci were consistent using MTAG. The present study underscores that multi-trait analysis in GWAS of true pleiotropic traits in relatively small sample sizes is a powerful approach to identify variants harboring correlated traits. Although these novel loci could not be directly associated with POAG it is likely that the genes in these regions mediate the glaucomatous process by their effect on the optic nerve morphology. For instance the *PLEKHG3* gene identified in this study is involved in the Rho signaling cascade, this pathway is known to play a crucial role in POAG pathophysiology and is currently targeted for new therapies for POAG^33^. Our bioinformatic analyses suggests that both the *PPP1R36-PLEKHG3* and *SERPINE3* variants are eQTL’s opening avenues to counteract the problem by RNA interference. Further research including exome sequencing and functional studies are needed to further define these genes in the mechanism of POAG.

## Methods

### Study design

We performed a meta-analysis of European origin GWAS’s imputed to HRC reference panel release 1. We analyzed five outcomes: CA, DA, VCDR, IOP, and CCT. The CA phenotype was adjusted for DA in all analyses since these phenotypes are clearly correlated (Pearson correlation coefficient 0.6). Subsequently, we performed multi-trait analysis for CA, DA, VCDR, IOP, and CCT. Replication was carried out for the single trait as well as the multi-trait analysis in a meta-analysis of 5 Asian cohorts imputed to 1000 genomes. We also tested significance of lead SNPs in three independent POAG cohorts.

### Study samples, phenotyping, and genotyping

All studies included in this meta-analysis are part of the International Glaucoma Genetics Consortium (IGGC). A description of the details of all cohorts participating in this study can be found in Supplementary Note and Supplementary Tables 1–6. The mean IOP, VCDR, CCT, CA, and DA of both eyes was used for the analyses. In case of missing or unreliable data for one eye, the measurement of the other eye was used instead. For subjects who received IOP-lowering medication, the measured IOP was multiplied by a factor of 1.3. The total number of individuals in the meta-analysis was 24,493 for CA, 24,509 for DA, 25,180 for VCDR, 31,269 for IOP, and 16,204 for CCT. All studies were performed with the approval of the local institutional review board (Supplementary Note) and written informed consent was obtained from all participants in accordance with the Declaration of Helsinki.

Genotyping was performed using commercially available Affymetrix or Illumina genotyping arrays (Supplementary Table 7). Quality control was executed independently for each study. To facilitate meta-analysis, each cohort performed genotype imputation using either the Sanger imputation service (https://imputation.sanger.ac.uk) or the Michigan imputation server (https://imputationserver.sph.umich.edu) with reference to the HRC panel, version 1 or 1.1^34^.

### Association analysis in discovery cohorts

Within each discovery cohort, each genotyped or imputed variant was tested for association with each of the traits, assuming an additive genetic model. The measurements were adjusted for sex, age, and five principal components in all cohorts and if necessary also for cohort-specific covariates (Supplementary Table 8). Family-based studies were adjusted for family structure. Given the clear correlation of CA with DA (Pearson’s correlation *r* = 0.59 in Rotterdam Study I), the CA GWAS was adjusted for DA in all discovery cohorts prior to meta-analysis. Linear regression was employed for studies with unrelated individuals, and linear mixed effects models were used to account for family structure in the family-based studies.

### Centralized quality control

Before meta-analysis, a centralized quality control procedure implemented in EasyQC was applied to individual study association summary statistics to identify outlying studies^35^. We included variants with imputation quality ≥ 0.3 (e.g., Minimac *R*^2^) and expected minor allele count >6. Additional checks for quality control were applied on the already filtered datasets including review of P–Z-plots, allele frequency plots and calculation of genomic inflation factor λ.

### Meta-analysis of discovery cohorts

The association results of all studies were combined in a fixed effect inverse variance meta-analysis in METAL^36^, since there was no sample overlap or cryptic relatedness as checked by LD score regression (see methods genetic overlap). This tool also applies genomic control by correcting the test statistics to account for small amounts of population stratification or unaccounted relatedness. We also assessed heterogeneity by calculating *I*^2^ values and Cochrans *Q*-test for heterogeneity as implemented in METAL. After meta-analyses of all available variants, we excluded the variants that were not present in at least three studies. This resulted in 11,830,838 variants for CA, 11,764,957 for DA, 11,901,698 variants for VCDR, 12,426,120 for IOP, and 9,249,813 variants for CCT. The remaining variants per trait were used to create Manhattan plots and QQ-plots, see Supplementary Figs. 4 and 5. The meta-analysis resulted in 1918 SNPs with a *p* value less than 5 × 10^−8^ for CA, 2029 for DA, 2473 for VCDR, 156 for IOP, and 1288 for CCT. Re-running the meta-analysis excluding TEST-BATS study to show that the significantly younger mean age in this study did not distorted our findings showed nearly perfect correlation between effect estimates from the full analysis and the effect estimates from in the analysis excluding TEST-BATS (CA *r* = 0.99; DA *r* = 0.99; VCDR = 0.99; IOP = 0.99). Furthermore, the mean differences between effect estimates found in the full analysis and the effect estimates found in the analysis excluding TEST-BATS were zero (CA mean difference = 0, SD = 0.00; DA mean difference = 0, SD = 0.00; VCDR mean difference = 0, SD = 0.01; IOP mean difference = 0, SD = 0.06), this also suggests that the younger age in the TEST-BATS study has not biased the results.

### Selection of independent variants

We examined whether multiple independent variants at a given locus influenced a trait and if they were independent of previous findings, we used the genome-wide complex trait analysis software (GCTA)^37^. This tool performs a stepwise selection procedure to select multiple associated SNPs by a conditional and joint (–CoJo) analysis approach using summary-level statistics from a meta-analysis and LD corrections between SNPs. The three Rotterdam Study cohorts (*N* = 5815), imputed with the HRC reference panel version 1, were used as the reference to calculate the LD, because it represents the largest discovery studies. LD was calculated between pairwise SNPs, but any SNP further than 10 Mb apart were assumed to not be in LD. All autosomal chromosomes were analyzed, with MAF restricted to ≥0.01 estimated from the three Rotterdam Study cohorts. The independent variants were annotated by Haploreg^19^, see Supplementary Table 9.

### Identification of potential novel variants

Previously, Springelkamp et al.^8,38,39^, Iglesias et al.^12^, Hysi et al.^40^, and Lu et al.^41^ identified various loci associated with CA, CCT, DA, IOP, and VCDR by GWAS with the HapMap and 1000 Genomes as a reference panel for imputations. To identify new variants, we investigated if any of the independent variants were within 1 Mb of a known loci identified for the same trait by Springelkamp et al.^8,38,39^, Iglesias et al.^12^, Hysi et al.^40^, and Lu et al.^41^. We created locuszoom plots and forest plots of all potential novel variants, see Supplementary Figs. 5 and 6. Variants showing significant association with a trait and are within 1 Mb of a previous identified locus were annotated to the known variant.

### Multi-trait analysis

For multi-trait genome-wide association analysis we applied the CPASSOC package developed by Zhu et al.^13^. We used CPASSOC for two analyses to combine the association results from CA, DA, VCDR, and from IOP and CCT. CPASSOC generates two statistics, SHom and SHet. SHom is similar to the fixed effect meta-analysis method but accounts for the correlation of summary statistics because of the correlated traits. SHom uses the sample size of a trait as a weight instead of variance, so that it is possible to combine traits with different measurement scales. SHet is an extension of SHom, but power can be improved when the genetic effect sizes are different for different traits. To compute statistics SHom and SHet, a correlation matrix is required to account for the correlation among traits or induced by overlapped or related samples from different cohorts. We followed the approach previously described by Park et al.^42^, to calculate this correlation matrix. Briefly, we used all independent SNPs (*r*^2^ < 0.2) present in datasets that were not associated with any of the traits (−1.96 > *Z* score < 1.96), and took the Pearson’s correlation of their *Z*-scores^13^. For both tests QQ-plots were created (Supplementary Fig. 8). Novel loci identified by CPASSOC (*p* < 5 × 10^−8^) that were not implicated in the single-trait analysis were validated using a second multi-trait method, MTAG. Similarly, MTAG also utilizes summary statistics as input, but performs LD score regression to estimate the genotypic and phenotypic variance-covariance matrices. In contrast to CPASSOC, MTAG performs association tests for each individual trait by boosting the power of a signal and providing an estimation of the underlying association via the multi-trait variance-covariance structure. We applied MTAG to SNPs MAF > 0.01 for combing the analysis of CA, DA, and VCDR, and the analysis of IOP and CCT. For the European sample we used the 1000Genomes European pre-calculated LD scores and for the Asians the 1000Genomes East-Asian pre-calculated LD scores (https://data.broadinstitute.org/alkesgroup/LDSCORE/). We then validated each of the genome-wide significant signals identified by CPASSOC in the MTAG results.

### Replication in Asian cohorts imputed to 1000Genomes

All independent SNPs identified with *p* < 5 × 10^−8^ in the discovery stage (single and multi-trait analysis) were carried forward for replication in Asians. For single-trait analyses, we validated these signals in fixed effect meta-analyses previously reported by Springelkamp et al. (CA, DA, VCDR, and IOP) and Iglesias et al. (CCT). Similar as in the discovery stage, we also performed a multi-trait CPASSOC and MTAG analysis of CA, DA, VCDR, and IOP, CCT in the Asians using the 1000Genomes summary statistics. Association replication was sought at nominal (*p* < 0.05) levels. A brief description of the cohorts participating in this study can be found in the Supplementary Note. Descriptive statistics, phenotyping methods, genotype, and 1000 Genomes phase I version 3 (March 2012) imputation quality and control has been described previously in Springelkamp et al.^8^ and Iglesias et al.^10^.

### Validation in POAG case–control studies

To evaluate whether SNPs identified in the European HRC discovery stage (*p* < 5 × 10^−8^) that replicated at nominal significance (*p* < 0.05) in Asians 1000Genomes have a shared component with primary open-angle glaucoma we validated these SNPs in three POAG case–control studies from NEIGBOHR/MEEI, Southampton and UK Biobank Eye and Vision Consortium. Phenotyping and genotyping methods are provided in Supplementary Note 1.3 and Supplementary Table 9. For the queried SNPs summary statistics from NEIGBOHR/MEEI and Southampton were combined in a fixed-effect and random-effect meta-analysis as implemented in Metasoft^43^. Statistical significant level was corrected for the number of queried SNPs by the Bonferroni method.

### The genetic overlap between CA, DA, VCDR, IOP, and CCT

To further investigate the genetic overlap among CA, DA, VCDR, CCT, and IOP we used the LD Score regression implemented in LDSC^44^ to examine the pattern of genetic correlations. The LD score for each SNP measures the amount of pairwise LD(*r*^2^) with other SNPs within 1-cM (centimorgan) windows based linkage disequilibrium. Bivariate LD score regression can estimate the extent to which two phenotypes share genetic variance.

Summary statistics of the five meta-analysis were formatted to LDSC input files, we followed quality control as implemented by the LDSC software package (https://github.com/bulik/ldsc). We used pre-calculated LD scores provided by the developers for each SNP using individuals of European ancestry from the 1000 Genomes project that are suitable for LD score analysis in European populations. SNP heritability estimates for all traits and genetic correlations were then calculated between the traits, see Supplementary Data 3 and Fig. 2.

### Bioinformatical annotation

Using the software HaploReg (version 4.1)^19^ and RegulomeDB v1.1^45^, we annotated the potential regulatory functions of the replicated GWAS SNPs and their proxies (*r*^2^ > 0.8, 1000 genomes CEU) based on epigenetic signatures. We examined whether these variants (GWAS SNPs and variants in LD with the GWAS SNPs) overlapped with regulatory elements including DNAse hypersensitive sites, histone modifications, and transcription factor-binding sites in human cell lines and tissues from the ENCODE Project and the Epigenetic Roadmap Project. We then used the RegulomeDB score to assess their potential functional consequence, as described previously^46^.

### Pathway analysis

We applied FUMA, which uses a three way gene-mapping strategy, to assign genome-wide significant SNPs to genes of interest. For positional mapping, SNPs in LD with the independent SNPs were mapped to genes using a window of 10 kb. eQTL mapping was performed by mapping SNPs to genes up to 1 Mb (cis-eQTL). eQTLs from all tissues available in GTEx v6^23^, Blood eQTL browser^47^, BIOS eQTL browser^48^, and BRAINEAC^49^ were selected for the mapping. Chromatin interaction was based on GSE87112 (Hi-C) database as implemented in FUMA. We explored possible biological functions by pathway analysis for all variants that reached genome wide significance in the discovery stage and were nominal significant in the Asian replication set. These 55 associated variants (ONH = 32, IOP = 3, CCT = 20) were assigned to genes by FUMA mapping strategies. Prioritized genes for ONH traits were highly overlapping and were combined to form a set of 295 unique genes for further functional annotation in FUMA. For IOP and CCT 11 and 116 genes were prioritized respectively. We further investigated the FUMA-mapped genes for enrichment using hypergeometric enrichment tests on pre-defined gene-sets derived from MsigDB and WikiPathways. *p* Values were corrected based on Bonferroni method for the number of tested gene-sets.

### Statistics and reproducibility

Software used for the data analysis of this study: METAL (https://genome.sph.umich.edu/wiki/METAL), EasyQC (www.genepi-regensburg.de/easyqc), GCTA (http://cnsgenomics.com/software/gcta/), FUMA (https://fuma.ctglab.nl/), LDSC (https://github.com/bulik/ldsc), CPASSOC (http://hal.case.edu/zhu-web/), and MTAG (https://github.com/omeed-maghzian/mtag). In the single trait analyses and the multi-trait analyses variants surpassing a *p* value less than 5 × 10^−8^ were considered genome-wide significant and followed up for replication in the Asian replication sample. Replication was defined as variants surpassing nominal significance level *p* value < 0.05. Variants were only considered novel when located >1 Mb away from a previously reported variant.

### Reporting summary

Further information on research design is available in the Nature Research Reporting Summary linked to this article.

## Supplementary information

Supplementary Information

Description of Additional Supplementary Files

Supplementary Data 1–14

Reporting Summary

## Data Availability

The genome-wide summary statistics that support the findings of this study will be made available via the NHGRI-EBI GWAS Catalog website (https://www.ebi.ac.uk/gwas/downloads/summarystatistics) upon publication.

## Code availability

No previously unreported custom computer code or mathematical algorithm was used to generate results central to the conclusions.
