# Supplementary material for: Multi-trait genome-wide association study identifies new loci associated with optic disc parameters
Source: Commun Biol. 2019 Nov 27;2:435. doi: 10.1038/s42003-019-0634-9 (PMC6881308; doi:10.1038/s42003-019-0634-9)
Supplement: Supplementary file 1 — Supplementary Information [file 42003_2019_634_MOESM1_ESM.pdf]

Supplementary Figures

Supplementary Figure 1. The comparison of the results of Springelkamp et al.[1] and Iglesias et al.[2] with the meta-analysis of the discovery cohorts.

Panel (A) shows the comparison of β values, panel (B) shows the zoom in on panel (A), panel (C) shows the comparison of MAF values and panel (D) shows the comparison of p-values. The red circles are the CA loci, the yellow circles are the CCT loci, the green circles are the DA loci, the blue loci are the IOP loci and the purple circles are the VCDR loci.

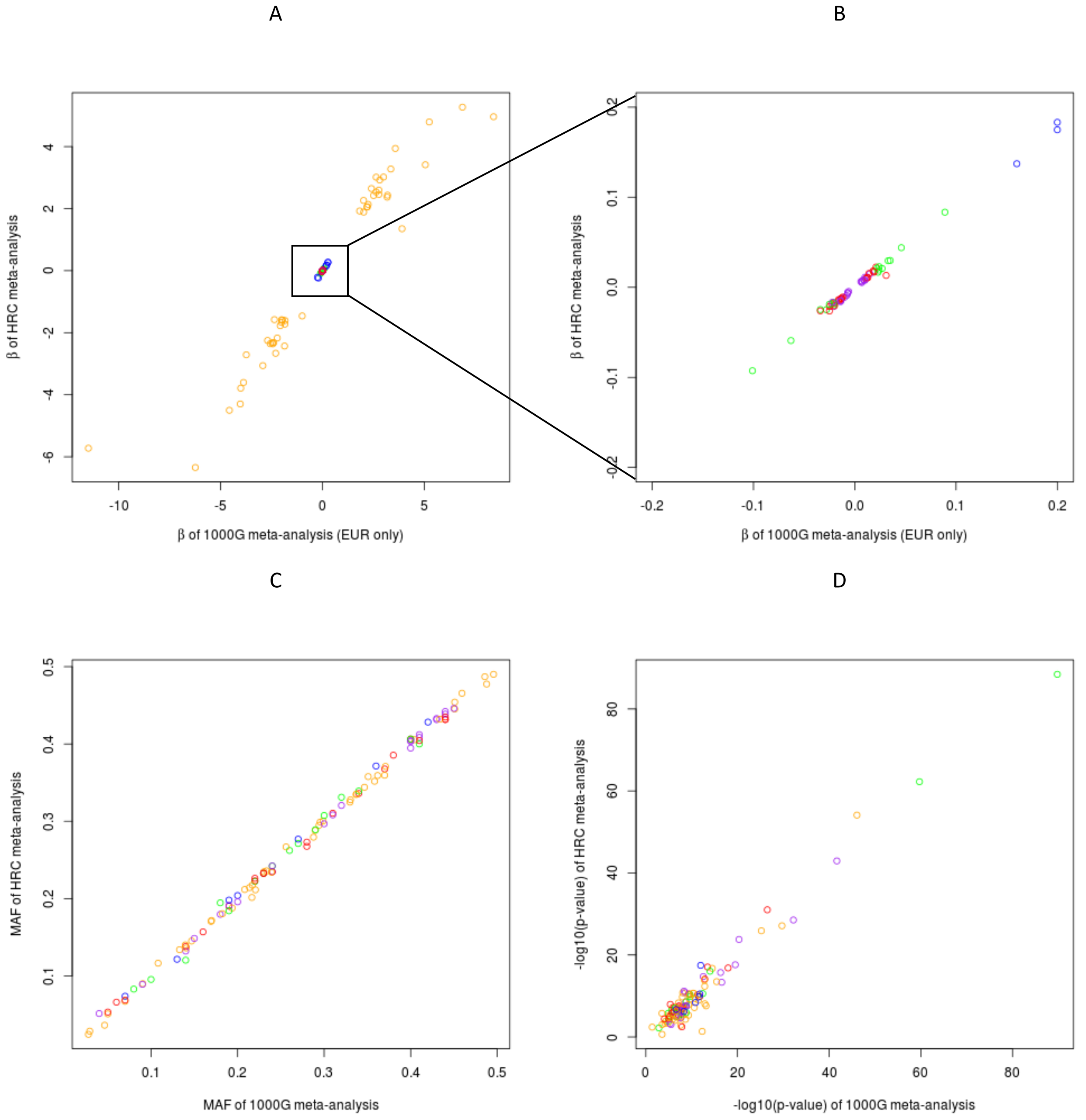

Supplementary Figure 2. Locuszoom plots of all novel identified variants for ONH by CPASSOC

rs551947583(SHet)

SNP not available in Locuszoom

rs11129176 (SHom)

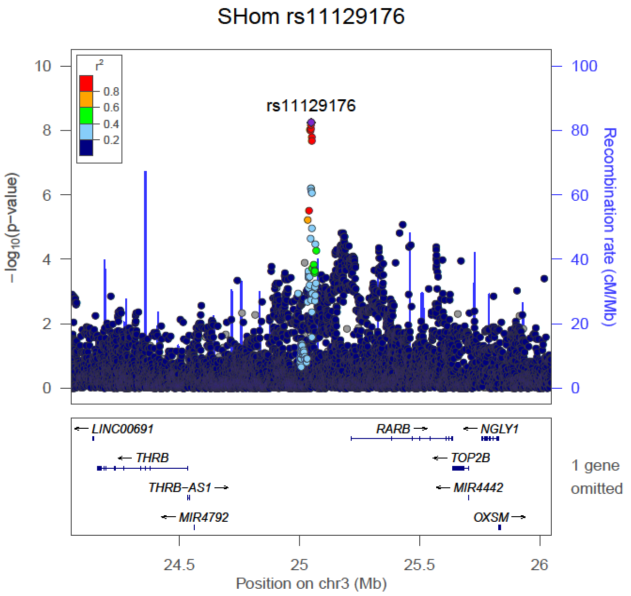

rs11129176 (SHet)

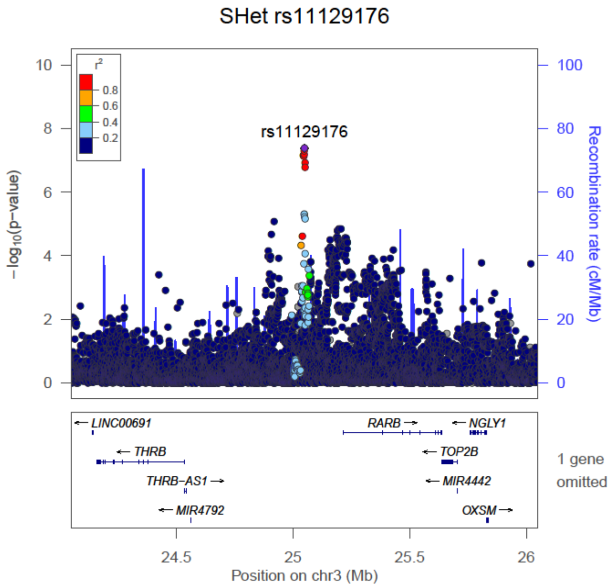

rs9471130 (SHom)

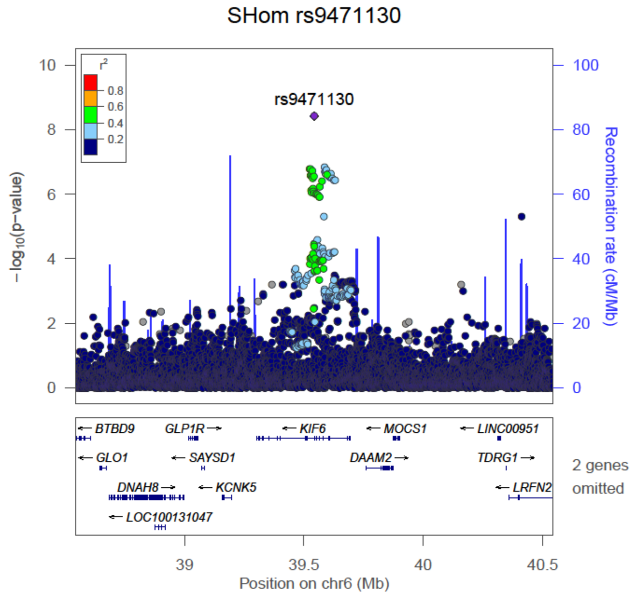

rs9471130 (SHet)

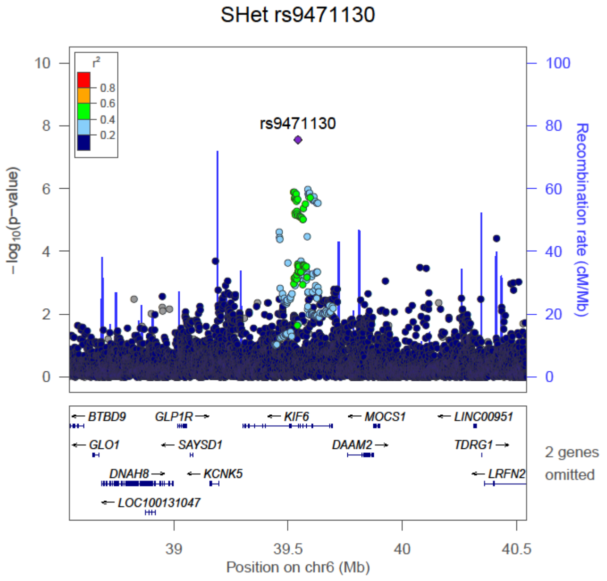

rs1681743 (SHom)

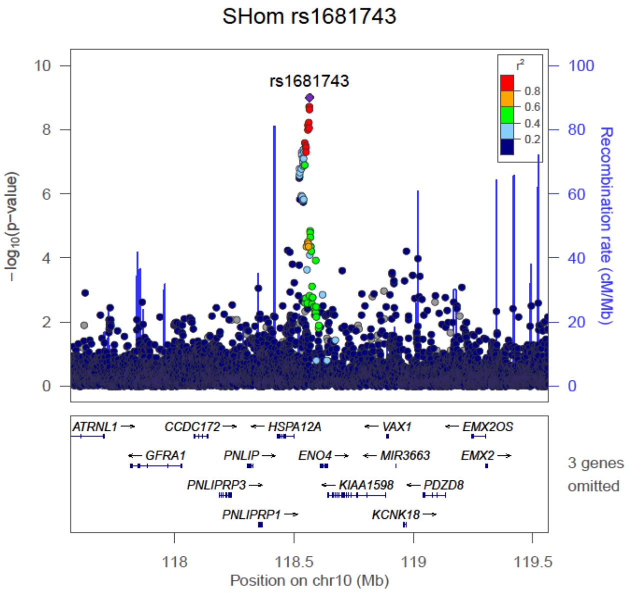

rs1681743 (SHet)

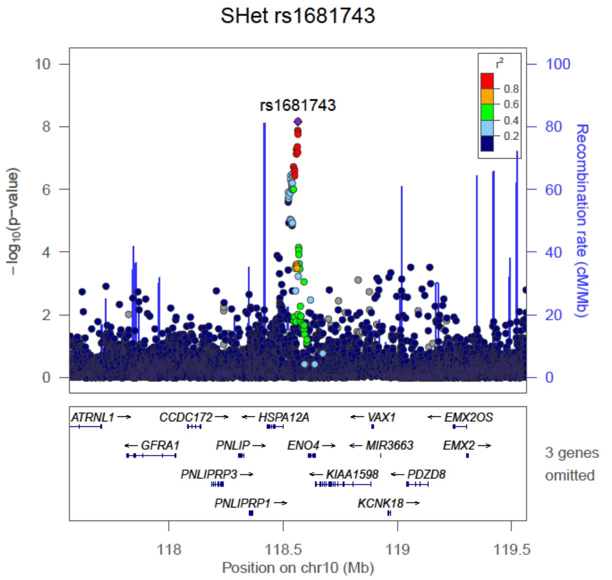

rs1028727

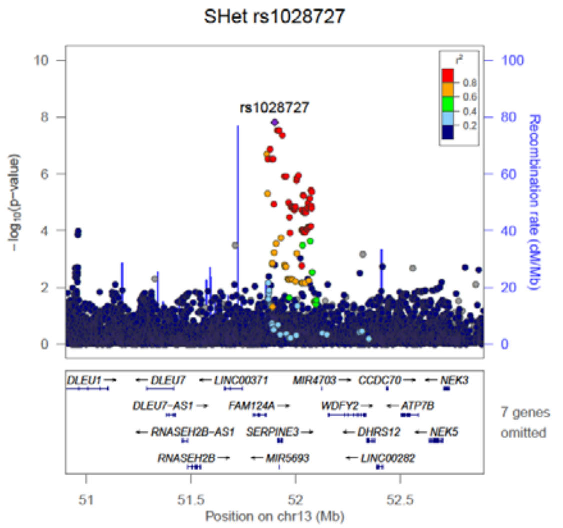

rs11158547 (SHom)

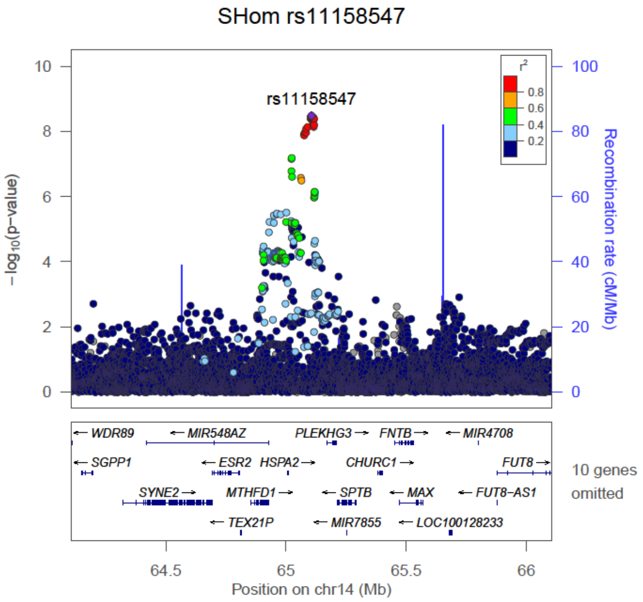

rs11158547 (SHet)

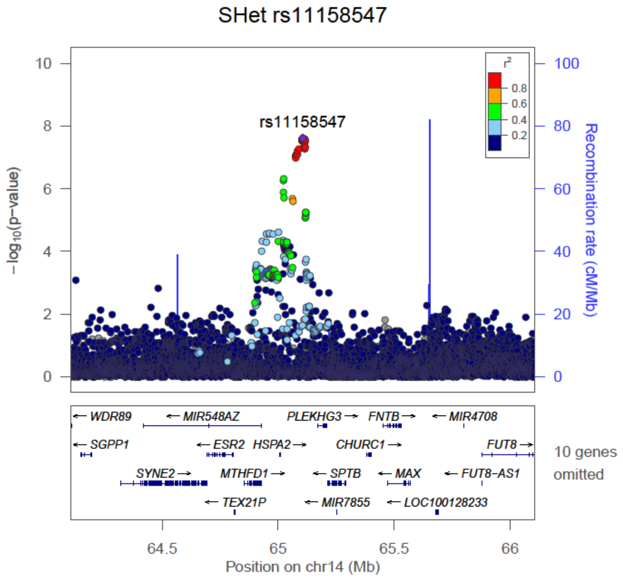

rs192647176 (SHom)

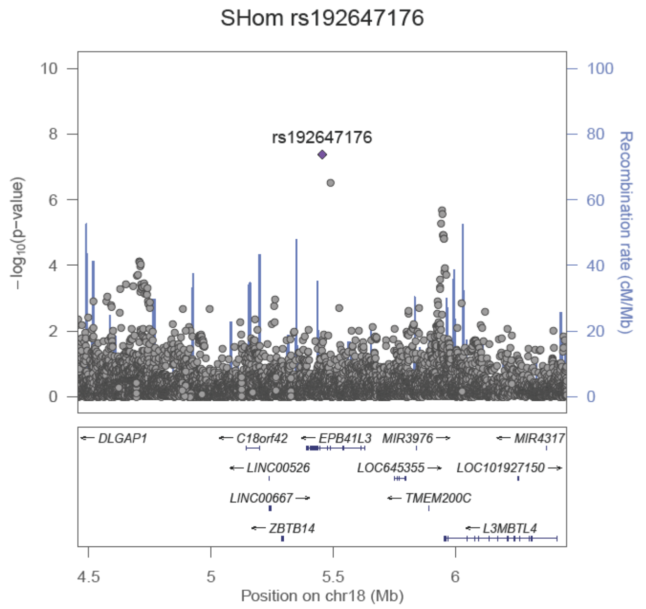
SHom rs192647176
rs192647176
−log10(p−value)
Recombination rate (cM/Mb)
0
2
4
6
8
10
0
20
40
60
80
100
DLGAP1
C18orf42
EPB41L3
MIR3976
MIR4317
LINC00526
LOC645355
LOC101927150
LINC00667
TMEM200C
ZBTB14
L3MBTL4
4.5
5
5.5
6
Position on chr18 (Mb)

Supplementary Figure 3. Circos plot depicting chromatin interactions and eQTL links for chromosome 14

Outer circle: regional association plots for SNPs *P*<0.05 SNPs in genomic risk loci are color-coded as a function of their maximum r2 to the one of the independent significant SNPs in the locus, as follows: red (r2 > 0.8), orange (r2 > 0.6), green (r2 > 0.4) and blue (r2 > 0.2). SNPs that are not in LD with any of the independent significant SNPs (with r2 ≤ 0.2) are grey. Second circle: positional map, genomic risk loci are highlighted in blue.
Third circle: mapped genes by chromatin interactions or eQTLs. Gene mapped only by chromatin interactions or only by eQTLs are colored orange or green, respectively. When the gene is mapped by both, it is colored red.

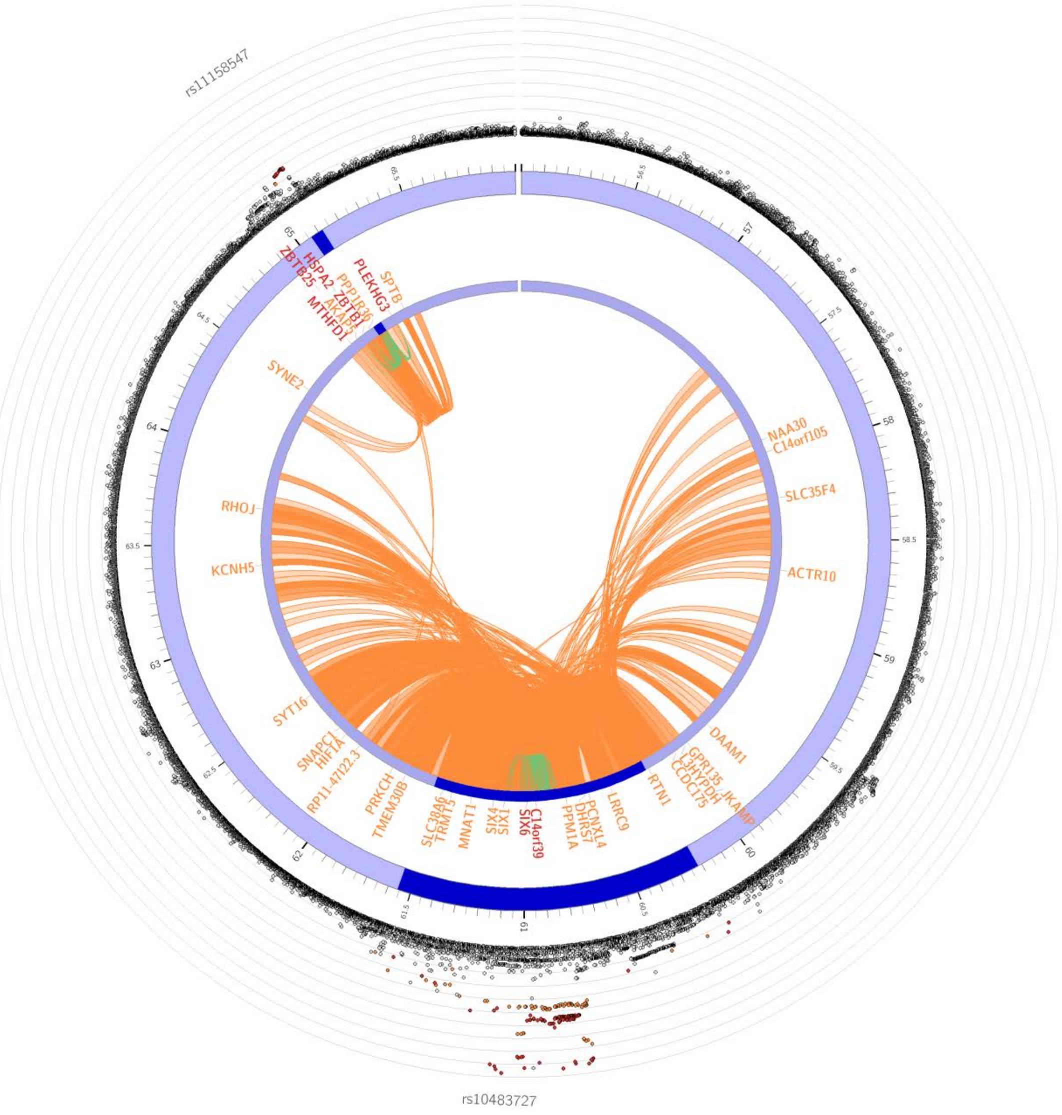

Supplementary Figure 3. Manhattan plots for all traits after the meta-analysis of all discovery cohorts.

Panel (A) shows the manhattan plot for CA, panel (B) shows the manhattan plot for CCT, panel (C) shows the manhattan plot for DA, panel (D) shows the manhattan plot for IOP, panel (E) shows the manhattan plot for VCDR. Variants that passed quality control and that were present in at least 3 cohorts were included. The black dotted line indicates the genome-wide significant line ($5 \cdot 10^{-8}$), the dark blue and the light blue dotes indicate the independent variants which are identified by GCTA within 1 Mb and more than 1 Mb away, respectively, from a published loci for that particular trait. As there are no published loci for RA, all independent loci identified by GCTA are considered new loci.

A

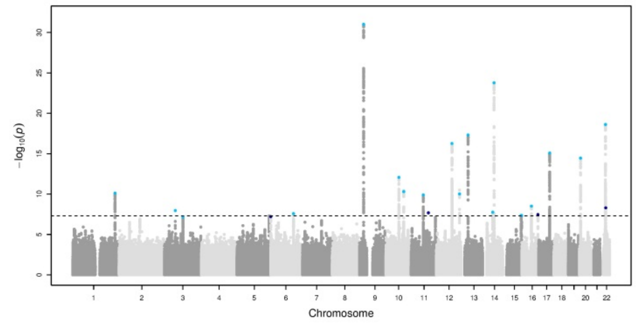

B

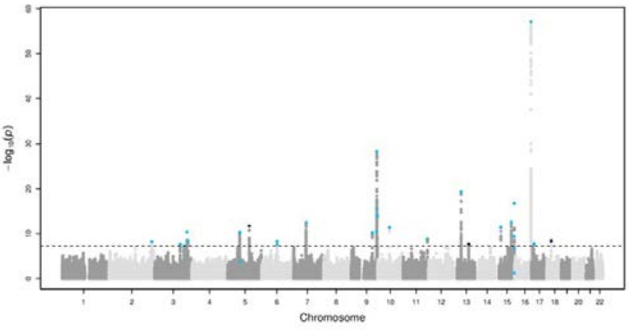

C

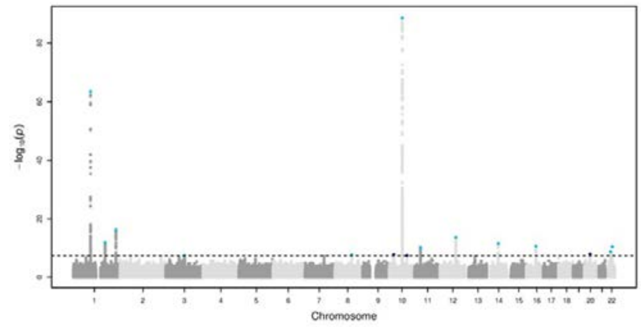

D

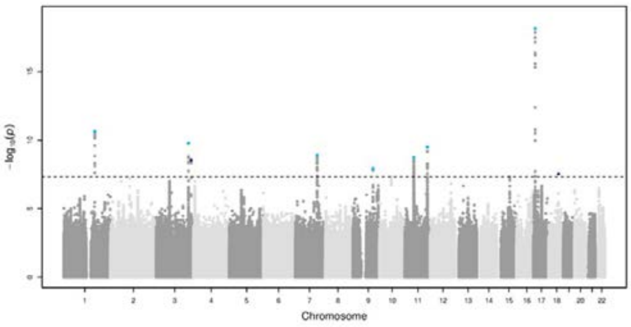

E

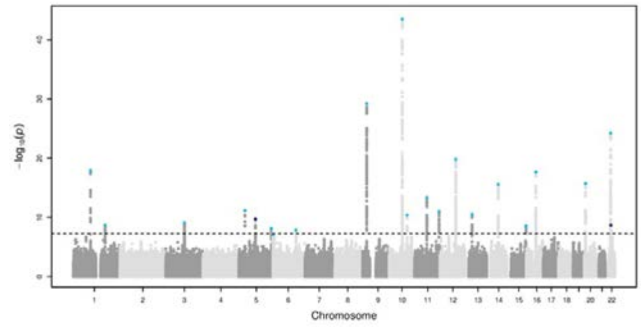

Supplementary Figure 4 QQ-plot for all traits after the meta-analysis of all discovery cohorts.

Panel (A) shows the manhattan plot for CA~DA, panel (B) shows the manhattan plot for CCT, panel (C) shows the manhattan plot for DA, panel (D) shows the manhattan plot for IOP, panel (E) shows the manhattan plot for VCDR. Variants that were present in at least 3 cohorts were included.

A

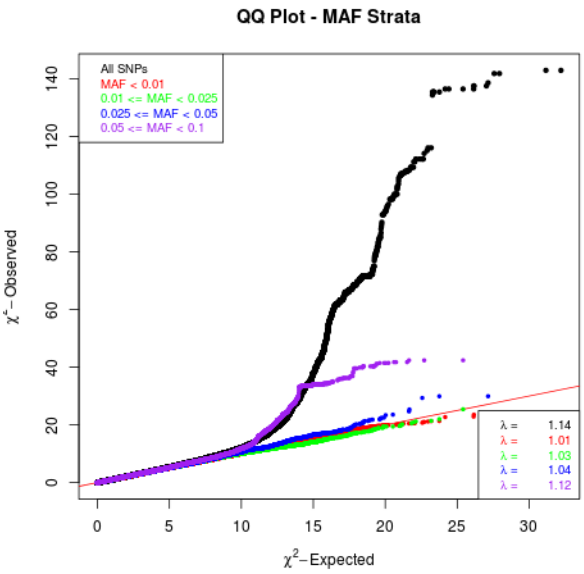

B

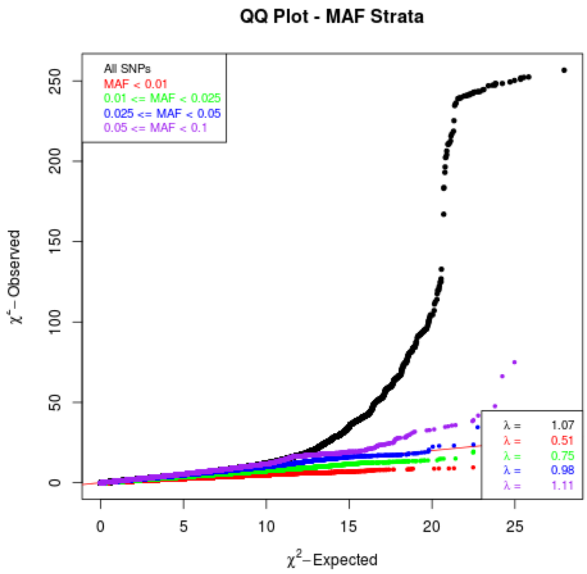

C

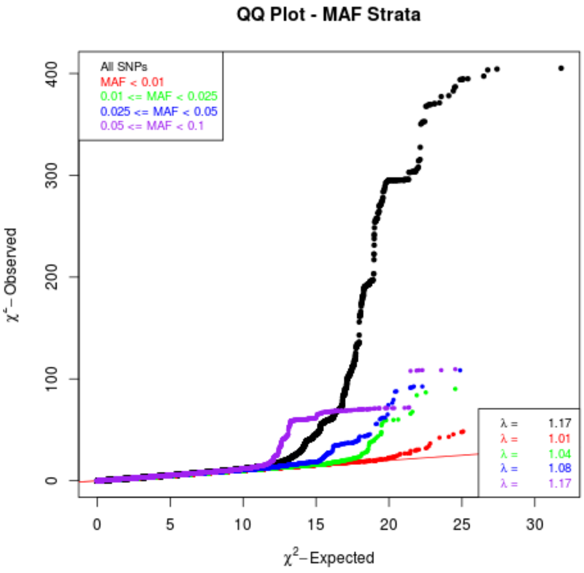

D

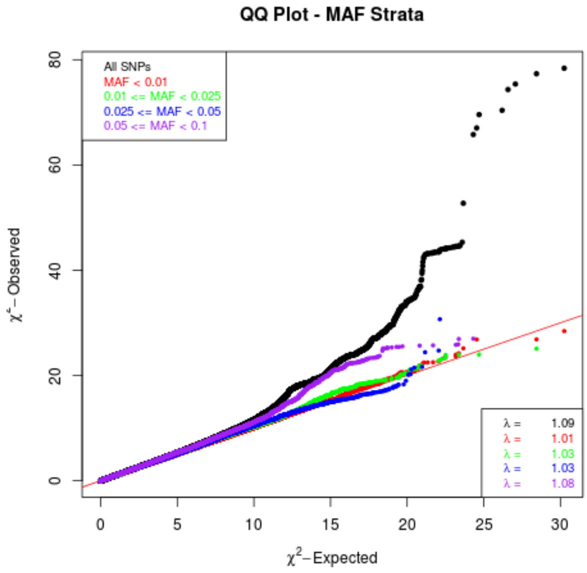

E

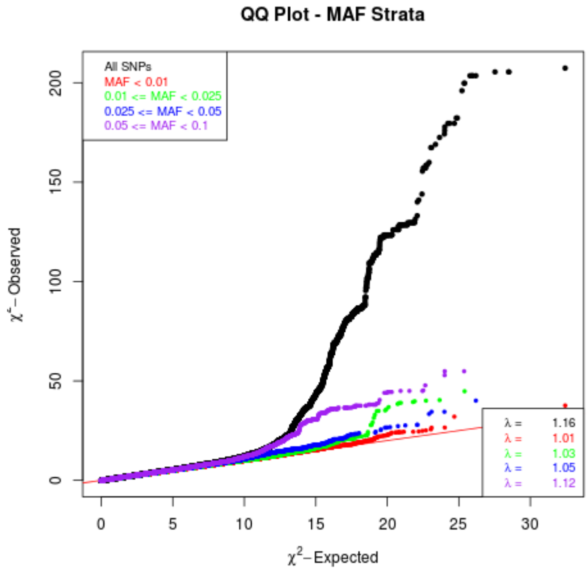

QQ Plot - MAF Strata
All SNPs
MAF < 0.01
0.01 <= MAF < 0.025
0.025 <= MAF < 0.05
0.05 <= MAF < 0.1
λ = 1.16
λ = 1.01
λ = 1.03
λ = 1.05
λ = 1.12
χ² – Observed
χ² – Expected

Supplementary Figure 5 Locuszoom plots of all novel identified variants within the single-trait meta-analysis.

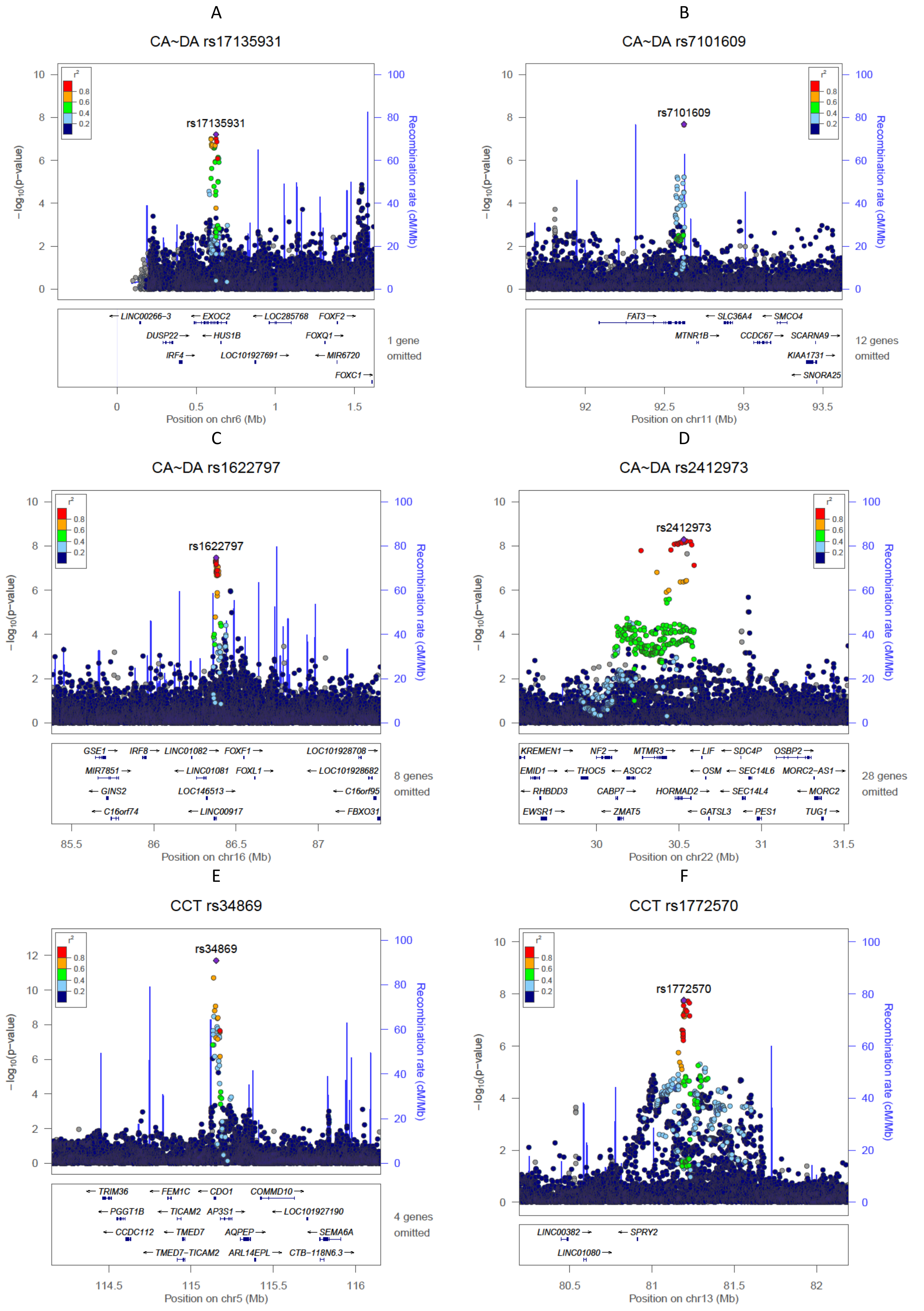

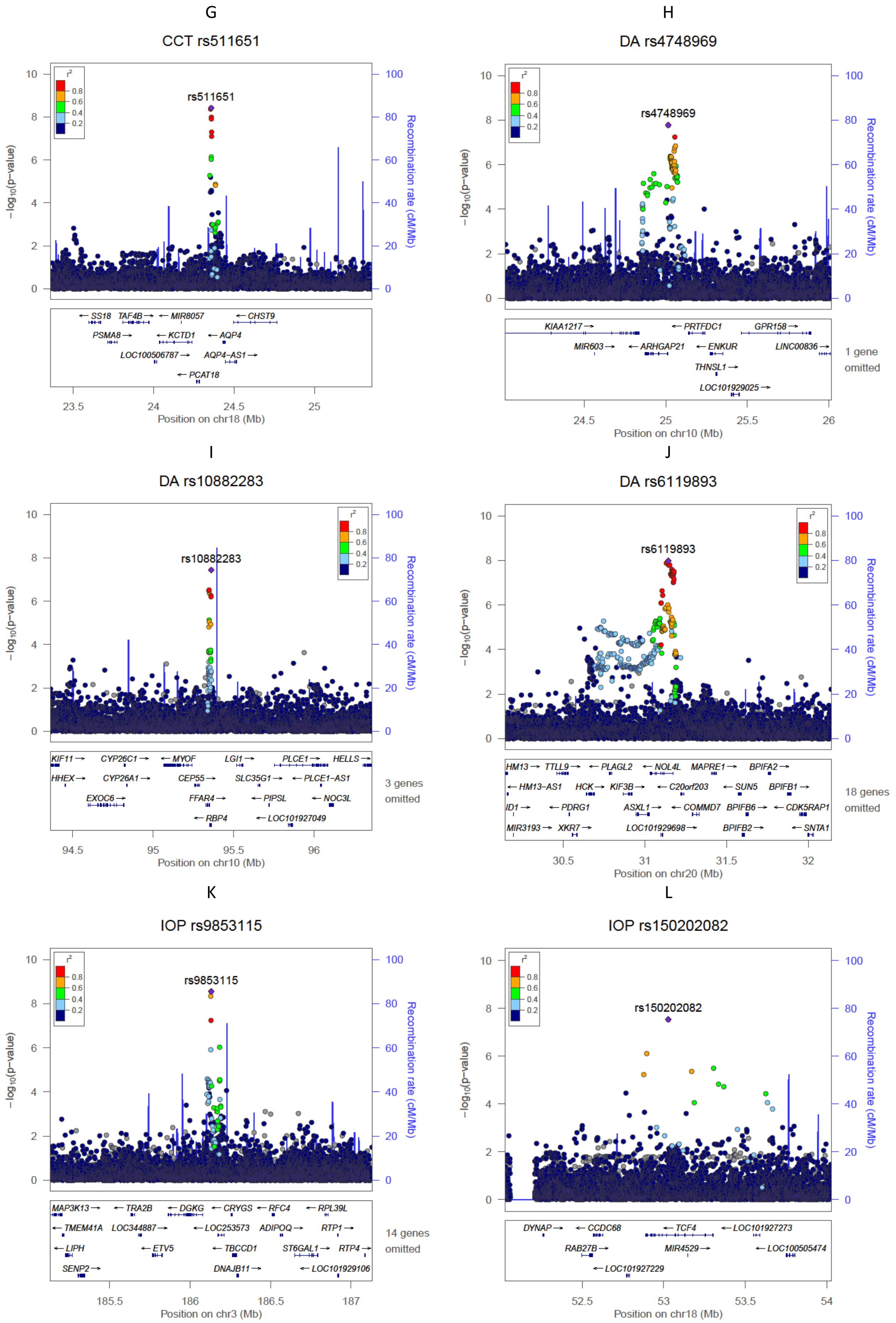
G
CCT rs511651
rs511651
SS18 TAF4B MIR8057 CHST9
PSMA8 KCTD1 AQP4
LOC100506787 AQP4-AS1
PCAT18
Position on chr18 (Mb)
H
DA rs4748969
rs4748969
KIAA1217 PRTFDC1 GPR158
MIR603 ARHGAP21 ENKUR LINC00836
THNSL1
LOC101929025
1 gene omitted
Position on chr10 (Mb)
I
DA rs10882283
rs10882283
KIF11 CYP26C1 MYOF LGI1 PLCE1 HELLS
HHEX CYP26A1 CEP55 SLC35G1 PLCE1-AS1
EXOC6 FFAR4 PIPSL NOC3L
RBP4 LOC101927049
3 genes omitted
Position on chr10 (Mb)
J
DA rs6119893
rs6119893
HM13 TTLL9 PLAGL2 NOL4L MAPRE1 BPIFA2
HM13-AS1 HCK KIF3B C20orf203 SUN5 BPIFB1
ID1 PDRG1 ASXL1 COMMD7 BPIFB6 CDK5RAP1
MIR3193 XKR7 LOC101929698 BPIFB2 SNTA1
18 genes omitted
Position on chr20 (Mb)
K
IOP rs9853115
rs9853115
MAP3K13 TRA2B DGKG CRYGS RFC4 RPL39L
TMEM41A LOC344887 LOC253573 ADIPOQ RTP1
LIPH ETV5 TBCCD1 ST6GAL1 RTP4
SENP2 DNAJB11 LOC101929106
14 genes omitted
Position on chr3 (Mb)
L
IOP rs150202082
rs150202082
DYNAP CCDC68 TCF4 LOC101927273
RAB27B MIR4529 LOC100505474
LOC101927229
Position on chr18 (Mb)
−log10(p-value)
Recombination rate (cM/Mb)
r2

M

VCDR rs115456027

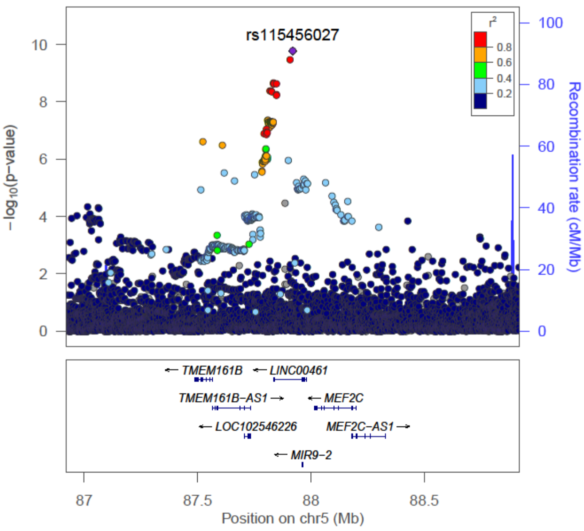

N

VCDR rs2412973

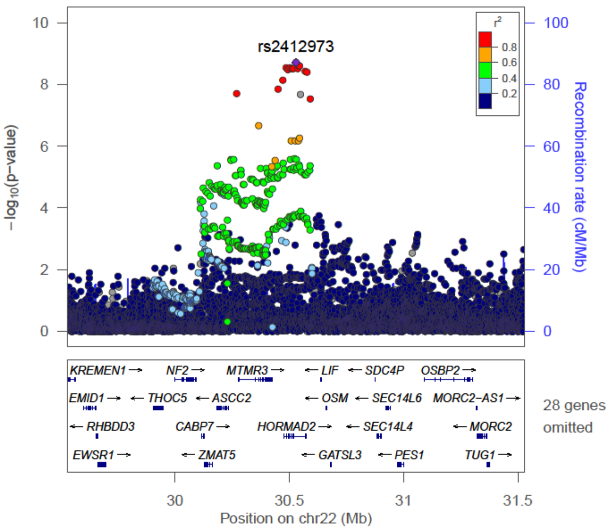

Supplementary Figure 6. Forest plots of all novel identified variants within the single-trait meta-analysis.

A

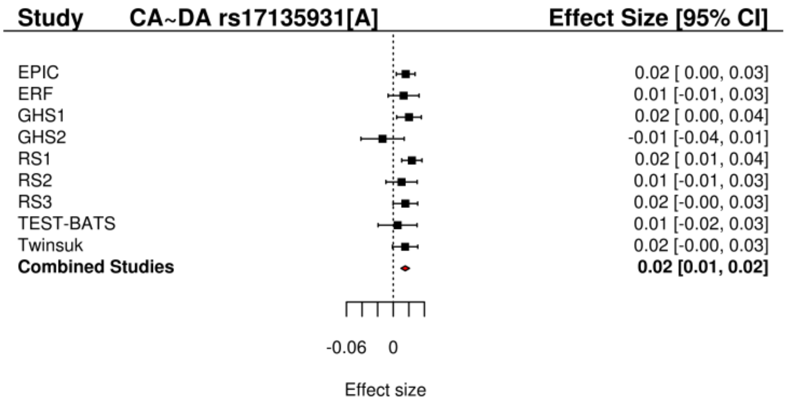

B

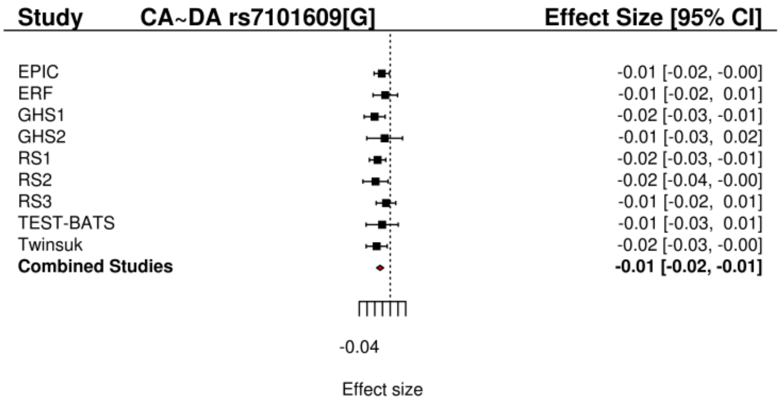

C

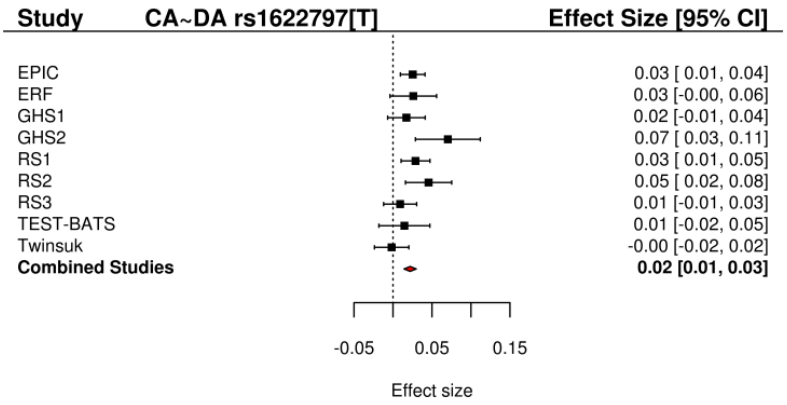

D

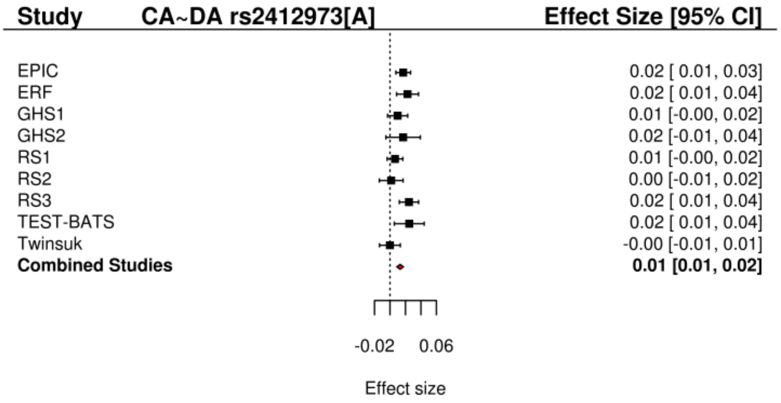
Study
CA~DA rs2412973[A]
Effect Size [95% CI]
EPIC
ERF
GHS1
GHS2
RS1
RS2
RS3
TEST-BATS
Twinsuk
Combined Studies
0.02 [0.01, 0.03]
0.02 [0.01, 0.04]
0.01 [-0.00, 0.02]
0.02 [-0.01, 0.04]
0.01 [-0.00, 0.02]
0.00 [-0.01, 0.02]
0.02 [0.01, 0.04]
0.02 [0.01, 0.04]
-0.00 [-0.01, 0.01]
0.01 [0.01, 0.02]
-0.02
0.06
Effect size

E

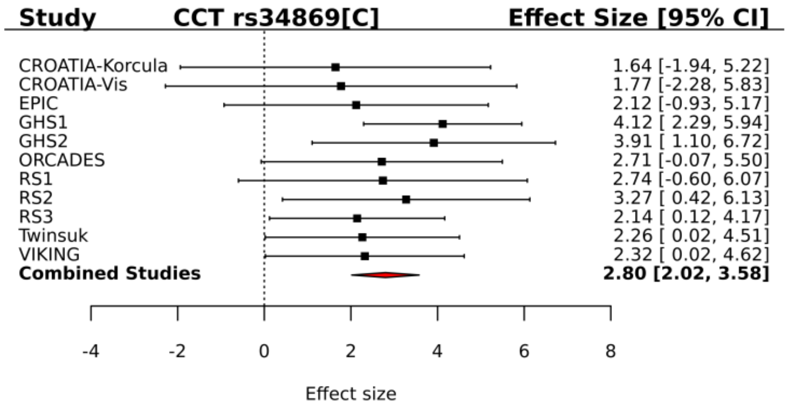
Study
CCT rs34869[C]
Effect Size [95% CI]
CROATIA-Korcula
CROATIA-Vis
EPIC
GHS1
GHS2
ORCADES
RS1
RS2
RS3
Twinsuk
VIKING
Combined Studies
1.64 [-1.94, 5.22]
1.77 [-2.28, 5.83]
2.12 [-0.93, 5.17]
4.12 [2.29, 5.94]
3.91 [1.10, 6.72]
2.71 [-0.07, 5.50]
2.74 [-0.60, 6.07]
3.27 [0.42, 6.13]
2.14 [0.12, 4.17]
2.26 [0.02, 4.51]
2.32 [0.02, 4.62]
2.80 [2.02, 3.58]
-4
-2
0
2
4
6
8
Effect size

F

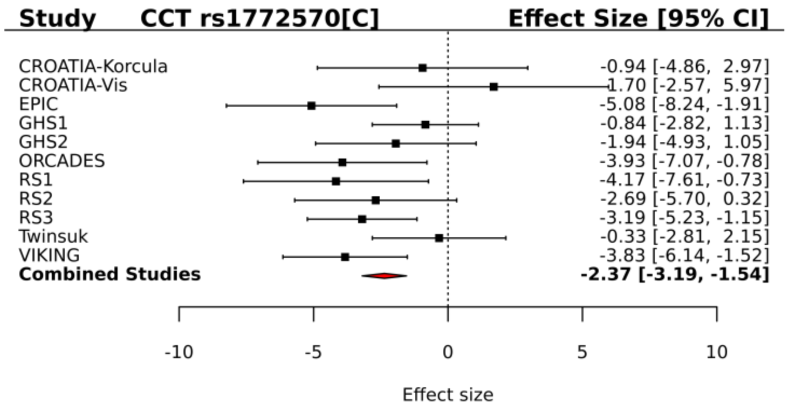
Study
CCT rs1772570[C]
Effect Size [95% CI]
CROATIA-Korcula
CROATIA-Vis
EPIC
GHS1
GHS2
ORCADES
RS1
RS2
RS3
Twinsuk
VIKING
Combined Studies
-0.94 [-4.86, 2.97]
1.70 [-2.57, 5.97]
-5.08 [-8.24, -1.91]
-0.84 [-2.82, 1.13]
-1.94 [-4.93, 1.05]
-3.93 [-7.07, -0.78]
-4.17 [-7.61, -0.73]
-2.69 [-5.70, 0.32]
-3.19 [-5.23, -1.15]
-0.33 [-2.81, 2.15]
-3.83 [-6.14, -1.52]
-2.37 [-3.19, -1.54]
-10
-5
0
5
10
Effect size

G

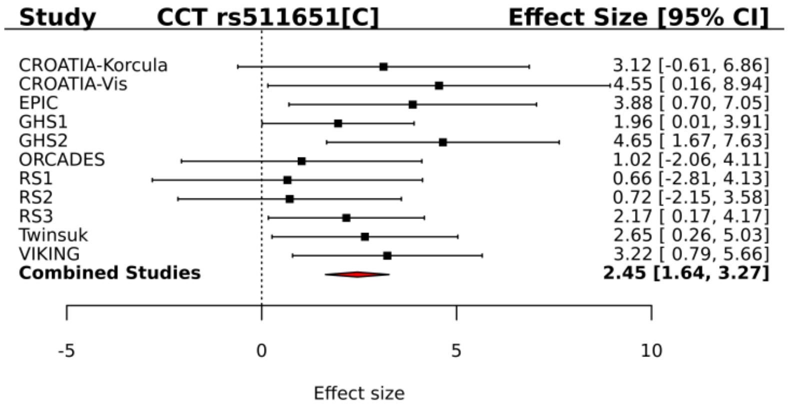
Study
CCT rs511651[C]
Effect Size [95% CI]
CROATIA-Korcula 3.12 [-0.61, 6.86]
CROATIA-Vis 4.55 [0.16, 8.94]
EPIC 3.88 [0.70, 7.05]
GHS1 1.96 [0.01, 3.91]
GHS2 4.65 [1.67, 7.63]
ORCADES 1.02 [-2.06, 4.11]
RS1 0.66 [-2.81, 4.13]
RS2 0.72 [-2.15, 3.58]
RS3 2.17 [0.17, 4.17]
Twinsuk 2.65 [0.26, 5.03]
VIKING 3.22 [0.79, 5.66]
Combined Studies 2.45 [1.64, 3.27]
-5
0
5
10
Effect size

H

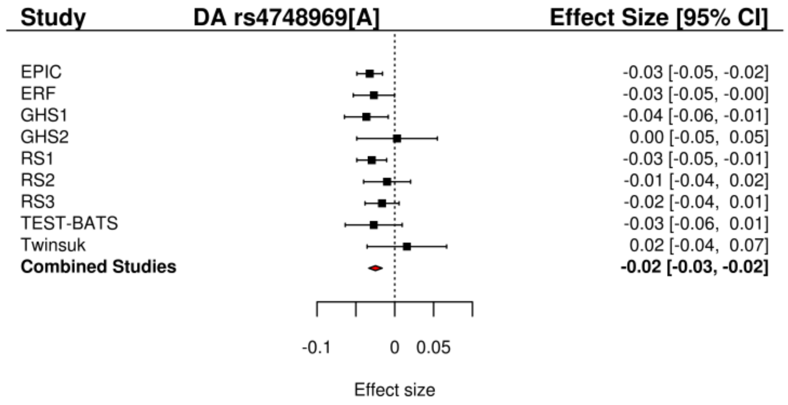
Study
DA rs4748969[A]
Effect Size [95% CI]
EPIC -0.03 [-0.05, -0.02]
ERF -0.03 [-0.05, -0.00]
GHS1 -0.04 [-0.06, -0.01]
GHS2 0.00 [-0.05, 0.05]
RS1 -0.03 [-0.05, -0.01]
RS2 -0.01 [-0.04, 0.02]
RS3 -0.02 [-0.04, 0.01]
TEST-BATS -0.03 [-0.06, 0.01]
Twinsuk 0.02 [-0.04, 0.07]
Combined Studies -0.02 [-0.03, -0.02]
-0.1
0
0.05
Effect size

I

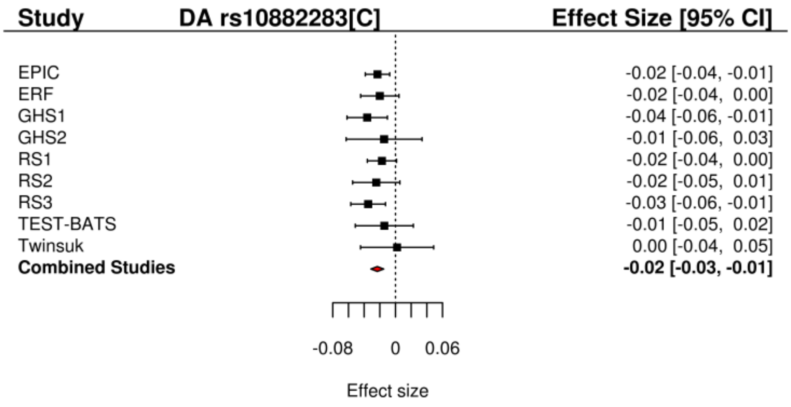
Study
DA rs10882283[C]
Effect Size [95% CI]
EPIC -0.02 [-0.04, -0.01]
ERF -0.02 [-0.04, 0.00]
GHS1 -0.04 [-0.06, -0.01]
GHS2 -0.01 [-0.06, 0.03]
RS1 -0.02 [-0.04, 0.00]
RS2 -0.02 [-0.05, 0.01]
RS3 -0.03 [-0.06, -0.01]
TEST-BATS -0.01 [-0.05, 0.02]
Twinsuk 0.00 [-0.04, 0.05]
Combined Studies -0.02 [-0.03, -0.01]
-0.08
0
0.06
Effect size

J

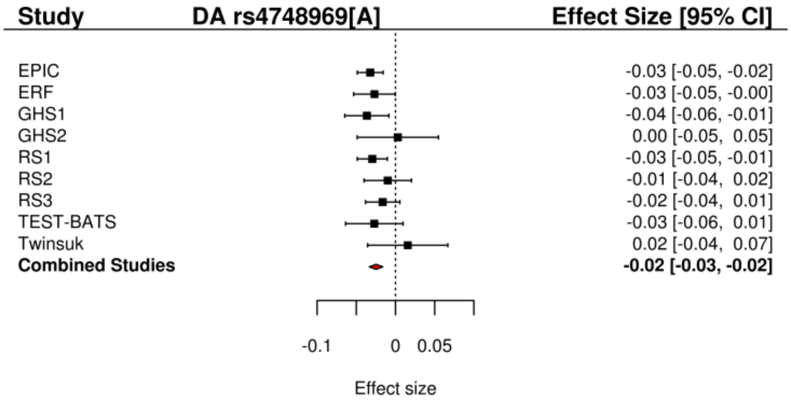
Study
DA rs4748969[A]
Effect Size [95% CI]
EPIC -0.03 [-0.05, -0.02]
ERF -0.03 [-0.05, -0.00]
GHS1 -0.04 [-0.06, -0.01]
GHS2 0.00 [-0.05, 0.05]
RS1 -0.03 [-0.05, -0.01]
RS2 -0.01 [-0.04, 0.02]
RS3 -0.02 [-0.04, 0.01]
TEST-BATS -0.03 [-0.06, 0.01]
Twinsuk 0.02 [-0.04, 0.07]
Combined Studies -0.02 [-0.03, -0.02]
-0.1
0
0.05
Effect size

K

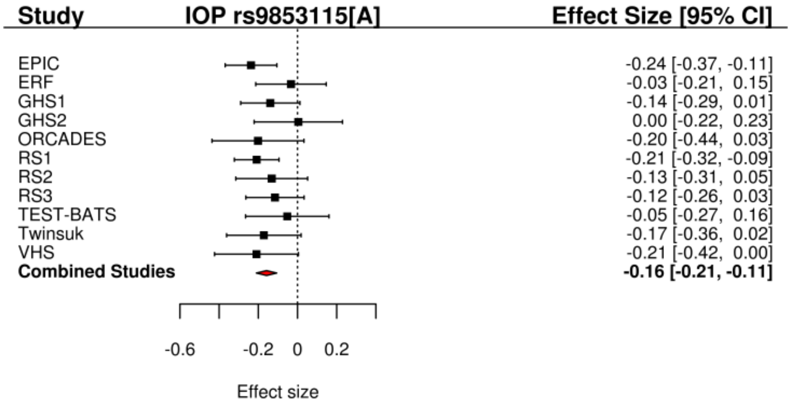
Study
IOP rs9853115[A]
Effect Size [95% CI]
EPIC -0.24 [-0.37, -0.11]
ERF -0.03 [-0.21, 0.15]
GHS1 -0.14 [-0.29, 0.01]
GHS2 0.00 [-0.22, 0.23]
ORCADES -0.20 [-0.44, 0.03]
RS1 -0.21 [-0.32, -0.09]
RS2 -0.13 [-0.31, 0.05]
RS3 -0.12 [-0.26, 0.03]
TEST-BATS -0.05 [-0.27, 0.16]
Twinsuk -0.17 [-0.36, 0.02]
VHS -0.21 [-0.42, 0.00]
Combined Studies -0.16 [-0.21, -0.11]
-0.6
-0.2
0
0.2
Effect size

L

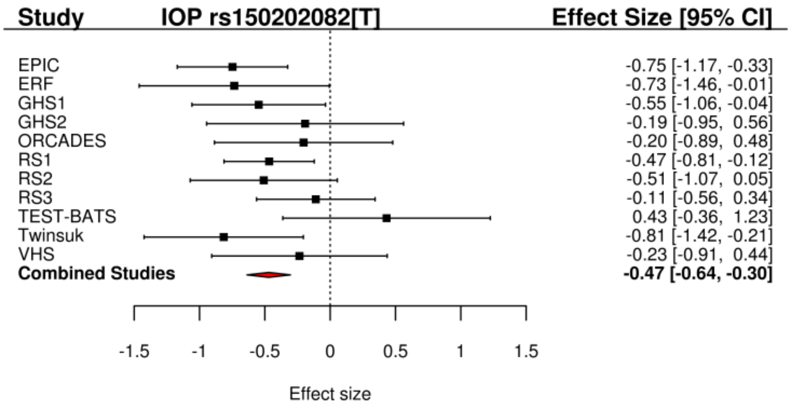
Study
IOP rs150202082[T]
Effect Size [95% CI]
EPIC -0.75 [-1.17, -0.33]
ERF -0.73 [-1.46, -0.01]
GHS1 -0.55 [-1.06, -0.04]
GHS2 -0.19 [-0.95, 0.56]
ORCADES -0.20 [-0.89, 0.48]
RS1 -0.47 [-0.81, -0.12]
RS2 -0.51 [-1.07, 0.05]
RS3 -0.11 [-0.56, 0.34]
TEST-BATS 0.43 [-0.36, 1.23]
Twinsuk -0.81 [-1.42, -0.21]
VHS -0.23 [-0.91, 0.44]
Combined Studies -0.47 [-0.64, -0.30]
-1.5
-1
-0.5
0
0.5
1
1.5
Effect size

M

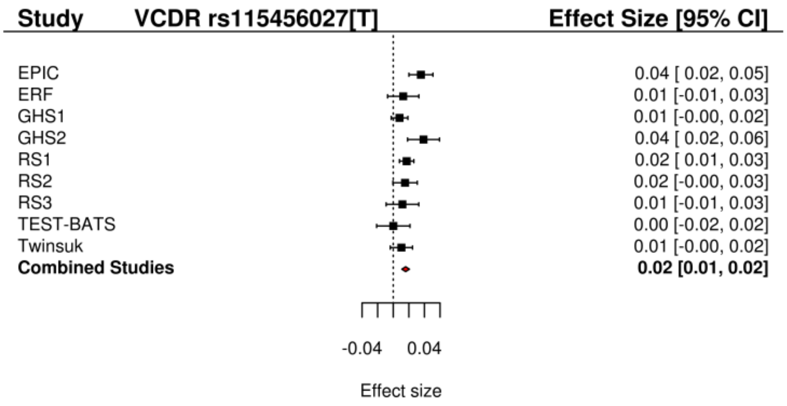
Study
VCDR rs115456027[T]
Effect Size [95% CI]
EPIC
ERF
GHS1
GHS2
RS1
RS2
RS3
TEST-BATS
Twinsuk
Combined Studies
0.04 [0.02, 0.05]
0.01 [-0.01, 0.03]
0.01 [-0.00, 0.02]
0.04 [0.02, 0.06]
0.02 [0.01, 0.03]
0.02 [-0.00, 0.03]
0.01 [-0.01, 0.03]
0.00 [-0.02, 0.02]
0.01 [-0.00, 0.02]
0.02 [0.01, 0.02]
-0.04
0.04
Effect size

N

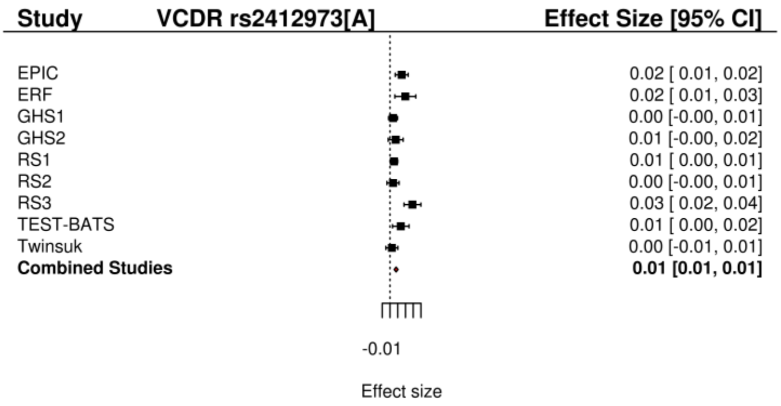
Study
VCDR rs2412973[A]
Effect Size [95% CI]
EPIC
ERF
GHS1
GHS2
RS1
RS2
RS3
TEST-BATS
Twinsuk
Combined Studies
0.02 [0.01, 0.02]
0.02 [0.01, 0.03]
0.00 [-0.00, 0.01]
0.01 [-0.00, 0.02]
0.01 [0.00, 0.01]
0.00 [-0.00, 0.01]
0.03 [0.02, 0.04]
0.01 [0.00, 0.02]
0.00 [-0.01, 0.01]
0.01 [0.01, 0.01]
-0.01
Effect size

Supplementary figure 7. plot CPASSOC multi-trait analysis for ONH and IOP,CCT.

Panel (A) shows the QQ-plot for SHom ONH, panel (B) shows the QQ-plot for SHet ONH, panel (C) shows the QQ-plot for SHom IOP,CCT panel (D) shows the QQ-plot for SHet IOP,CCT

A

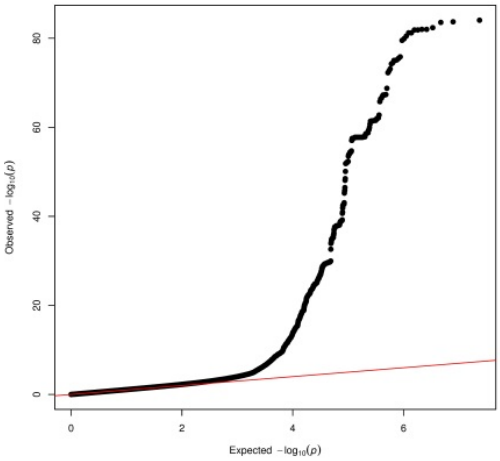

B

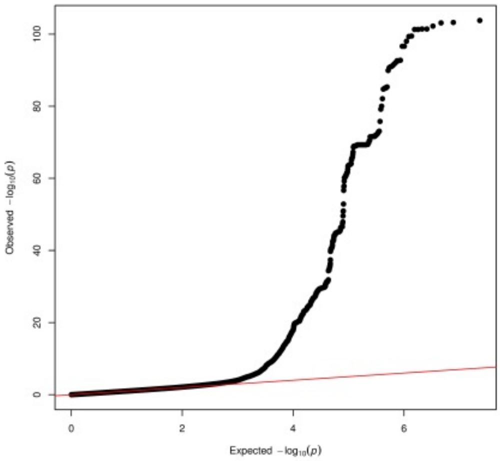

C

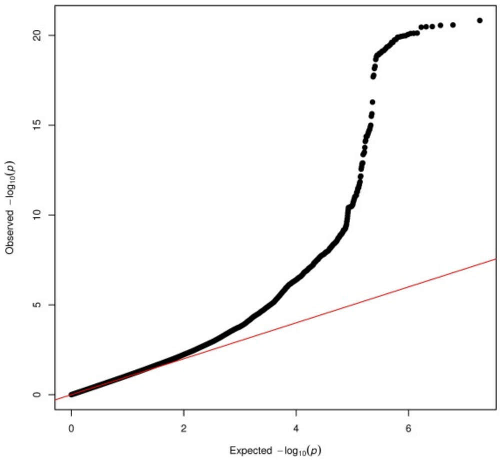

D

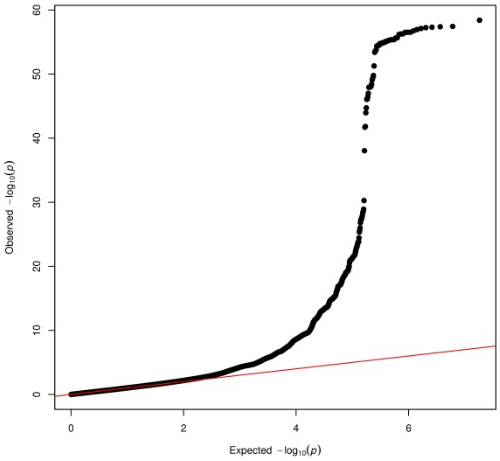

Supplementary Tables

Supplementary Table 1. Baseline characteristics: clinical characteristics.

GAT = Goldmann applanation tonometry. NA = not applicable

Study	Measurement method IOP	Measurement method CA, DA, RA and VCDR	Measurement method CCT
CROATIA-Korcula	NA	NA	Nidek hand held Echoscan US-1800 A- scan
CROATIA-Vis	NA	NA	Nidek hand held Echoscan US-1800 A- scan
EPIC-Norfolk Eye Study	Ocular Response Analyzer (ORA, Reichert, New York, USA; software V.3.01)	HRT3	Pachmate DGH 55, mean of 10 readings per eye; DGH Technology, Exton, PA
ERF	Goldmann applanation tonometry (Haag-Streit, Bern, Switzerland)	Heidelberg Retina Tomograph 2, Heidelberg Engineering, Heidelberg, Germany	NA
GHS1	Noncontact Tonometer (Nidek NT-2000™, Nidek Co., Japan)	Visucam PRO NM™ and Visupac™ (Carl Zeiss Meditec AG, Jena, Germany)	Pachycam™ (Oculus, Wetzlar, Germany)
GHS2	Noncontact Tonometer (Nidek NT-2000™, Nidek Co., Japan)	Visucam PRO NM™ and Visupac™ (Carl Zeiss Meditec AG, Jena, Germany)	Pachycam™ (Oculus, Wetzlar, Germany)
ORCADES	Tonopen	NA	IOPac hand held pachymeter (Heidelberg engineering)
RS-I	Goldmann applanation tonometry (Haag-Streit, Bern, Switzerland)	ImageNet and stereoscopic fundus camera (Topcon TRC-SS2; Tokyo Optical Co., Tokyo, Japan)	Non-contact biometer Lenstar LS900, Haag-Streit, Köniz, Switerzerland
RS-II	Goldmann applanation tonometry (Haag-Streit, Bern, Switzerland)	ImageNet and stereoscopic fundus camera (Topcon TRC-SS2; Tokyo Optical Co., Tokyo, Japan)	Non-contact biometer Lenstar LS900, Haag-Streit, Köniz, Switerzerland
RS-III	Goldmann applanation tonometry (Haag-Streit, Bern, Switzerland)	Heidelberg Retina Tomograph 2, Heidelberg Engineering, Heidelberg, Germany	Non-contact biometer Lenstar LS900, Haag-Streit, Köniz, Switerzerland
TEST-BATS	TONO-PEN XL (Reichert, Inc., New York, USA) and Goldmann applanation tonometry (Haag-Streit, Bern, Switzerland)	Nidek 3-Dx fundus camera with custom planimetric software	ultrasound pachymetry using Tomey SP 2000 (Tomey Corp., Nagoya, Japan)
TwinsUK	Ocular Response Analyzer	Nidek 3-Dx fundus camera with custom planimetric software	Ultrasound pachymetry device
VIKING	Tonopen	NA	IOPac hand held pachymeter (Heidelberg engineering)

Supplementary Table 2. Baseline characteristics for all participating cohorts for the CA analysis.

The unit of CA is mm2.

Study	*N*	Mean age (sd)	Age range	% Men	Mean CA (sd)	CA range
EPIC-Norfolk Eye Study	5853	67.8 (7.7)	48-90	44.60	0.45 (0.33)	0.00-1.81
ERF	2018	48.2 (13.8)	18-85	44.00	0.43 (0.31)	0.00-2.10
GHS1	2372	55.5 (10.7)	35-74	47.85	0.55 (0.34)	0.07-2.63
GHS2	782	54.9 (10.8)	36-74	49.87	0.53 (0.33)	0.07-2.65
RS-I	5555	68.0 (8.4)	55-99	40.90	0.61 (0.34)	0.01-1.98
RS-II	1979	64.7 (7.7)	55-96	46.00	0.57 (0.32)	0.03-1.94
RS-III	2870	57.2 (6.6)	46-90	43.80	0.40 (0.30)	0.00-1.90
TwinsUK	1727	57.1(11.2)	16-83	1.50	0.31(0.24)	0.00-1.45
TEST-BATS	1356	20.4 (9.8)	5-79	45.00	0.45 (0.28)	0.01-1.47

Supplementary Table 3. Baseline characteristics for all participating cohorts for the CCT analysis.

The unit of CCT is μm.

Study	*N*	Mean age (sd)	Age range	% Men	Mean CCT (sd)	CCT range
CROATIA-Korcula	858	56.43(13.72)	18-98	36.00	557.49(35.30)	468-697
CROATIA-Vis	597	56.2 (14.2)	18-86	39.97	560.9 (34.3)	441-662
EPIC-Norfolk Eye Study	1178	70.9 (8.0)	49-92	50.68	555.0 (37.1)	451-680
GHS1	2822	55.9 (10.9)	35-74	48.87	551.2 (35.0)	440-676
GHS2	1133	55.1 (10.8)	36-74	50.04	562.1 (34.0)	436-681
ORCADES	1113	55.1 (14.3)	18-89	37.83	538.0 (32.7)	432-663
RS-I	873	76.3 (6.7)	56-96	47.88	544.4 (33.9)	432-679
RS-II	1215	72.7 (5.3)	65-98	46.50	547.7 (34.2)	433-669
RS-III	2391	62.3 (5.8)	51-94	43.50	550.3 (33.9)	404-691
TwinsUK	2163	56.5 (11.8)	16-83	2.26	545.2 (35.0)	369-658
VIKING	1888	49.7 (15.1)	18-91	40.04	541.9 (33.9)	410-680

Supplementary Table 4. Baseline characteristics for all participating cohorts for the DA analysis.

The unit of DA is mm2.

Study	*N*	Mean age (sd)	Age range	% Men	Mean DA (sd)	DA range
EPIC-Norfolk Eye Study	5856	67.8 (7.7)	48-90	44.70	1.86 (0.40)	0.60-3.51
ERF	2004	48.2 (13.8)	18-85	44.00	1.91 (0.35)	1.07-3.95
GHS1	2372	55.5 (10.7)	35-74	47.85	2.32 (0.45)	1.26-4.65
GHS2	782	54.9 (10.8)	36-74	49.87	2.34 (0.47)	1.07-4.85
RS-I	5563	68.0 (8.4)	55-99	41.00	2.42 (0.47)	0.58-5.13
RS-II	1983	64.7 (7.7)	55-96	46.10	2.33 (0.46)	1.13-5.19
RS-III	2872	57.2 (6.6)	46-90	43.80	1.92 (0.40)	0.75-4.22
TEST-BATS	1356	20.4 (9.8)	5-79	45.00	2.07 (0.39)	1.15-3.56
TwinsUK	1732	57.1 (11.2)	16-83.	1.50	2.59 (0.64)	0.59-5.31

Supplementary Table 5. Baseline characteristics for all participating cohorts for the IOP analysis.

The unit of IOP is mmHg.

Study	*N*	Mean age (sd)	Age range	% Men	Mean IOP (sd)	IOP range
EPIC-Norfolk Eye Study	6472	68.8 (8.0)	48-92	45.22	16.2 (3.8)	5.9-39.8
ERF	2462	49.9 (14.0)	17-87	44.52	15.2 (3.0)	6.5-25.4

GHS1	2720	55.5 (10.8)	35-74	48.67	14.2 (2.8)	7.4-35.8
GHS2	1112	54.9 (10.8)	36-74	50.27	13.9 (2.7)	5.9-27.3
ORCADES	1112	55.1 (14.4)	18-89	37.77	15.0 (2.7)	7.5-29.0
RS-I	6010	69.2 (9.0)	55-101	40.30	14.7 (3.2)	5.0-28.6
RS-II	2095	64.8 (7.9)	55-95	45.90	14.2 (3.1)	7.0-31.5
RS-III	2992	57.2 (6.8)	46-97	43.70	13.6 (2.9)	4.5-30.0
TEST-BATS	1798	22.1 (12.0)	5-81	44.00	15.8 (3.0)	6.0-26.0
TwinsUk	2510	60.0 (11.6)	16-85	2.20	15.6 (3.3)	5.4-33.0
VIKING	1986	49.99 (15.2)	18-91	40.23	14.8 (3.2)	5.0-30.6

Supplementary Table 6. Baseline characteristics for all participating cohorts for the VCDR analysis.

The unit of VCDR is ratio.

Study	*N*	Mean age (sd)	Age range	% Men	Mean VCDR (sd)	VCDR range
EPIC-Norfolk Eye Study	5866	67.8 (7.7)	48-90	44.70	0.34 (0.23)	0.00-1.00
ERF	2008	48.2 (13.8)	18-85	44.00	0.31 (0.20)	0.00-0.87
GHS1	2776	55.8 (10.9)	35-74	48.85	0.44 (0.11)	0.19-0.81
GHS2	1036	54.8 (10.9)	36-74	49.42	0.43 (0.11)	0.19-0.80
RS-I	5573	68.0 (8.4)	55-99	40.90	0.50 (0.13)	0.05-0.87
RS-II	1987	64.7 (7.7)	55-96	46.10	0.50 (0.13)	0.10-0.86
RS-III	2873	57.2 (6.6)	46-90	43.90	0.29 (0.21)	0.00-1.00
TEST-BATS	1330	20.3 (9.7)	5-79	45.00	0.45 (0.13)	0.08-0.88
TwinsUK	1731	57.1 (11.2)	16-83	1.50	0.34(0.11)	0.04-0.70

Supplementary Table 7. SNP genotyping and imputation details of the population based-studies

MAF = minor allele frequency; HWE = Hardy-Weinberg equilibrium

Study	Genotype platform	Pre imputation QC tresholds	Post imputation QC tresholds	HRC version
CROATIA-Korcula	Mix: Illumina CNV370v1,CNV370-Quadv3	MAF > 0.01; SNPcallrate >0.98; HWE> 1x10E-6	Monomorphic, duplicate and triallelic removed ; imputation quality-INFO ≥0.4	HRC r1
CROATIA-Vis	IlluminaHumanHap300v1	MAF > 0.01; SNPcallrate >0.98; HWE> 1x10E-6	Monomorphic, duplicate and triallelic removed ; imputation quality-INFO ≥0.4	HRC r1
EPIC-Norfolk Eye study	Affymetrix UK Biobank Axiom Array	SNP exclusion criteria included: call rate < 95%, abnormal cluster pattern on visual inspection, plate batch effect evident by significant variation in minor allele frequency, and/or Hardy-Weinberg equilibrium P < 10-7. Sample exclusion criteria included: DishQC < 0.82 (poor fluorescence signal contrast), sex discordance, sample call rate < 97%, heterozygosity outliers (calculated separately for SNPs with minor allele frequency >1% and <1%), rare	exclusion: info<0.3, or monomorphic and singletons	HRC r1

		allele count outlier, and impossible identity-by-descent values. Following these exclusions, there were no ethnic outliers.		
ERF	Illumina 6k, Illumina 318K, Illumina 370K and Affymetrix 250K	Sample QC: Sample callrate >97.5%, no excess autosomal heterozygosity (<0.336), no sex-mismatch. SNPs QC: SNP call_rate >98% MAF >0.01, HWE> 1x10E-6	MAF ≥ 0,001 ; imputation quality ≥0,3	HRC r1
GHS1	Affymetrix SNP Array 6.0	None	MAF ≥ 0,001 ; imputation quality ≥0,3	HRC r1.1
GHS2	Affymetrix SNP Array 6.0	none	MAF ≥ 0,001 ; imputation quality ≥0,3	HRC r1.1
ORCADES	Mix: IlluminaHumanHap300v2, HumanCNV370-Quad and HumanOmniExpress-12v1	MAF > 0.01; SNPcallrate >0.98; HWE> 1x10E-6	Monomorphic, duplicate and triallelic removed ; imputation quality-INFO ≥0.4	HRC r1
RS-I	illumina 550 (+duo) Illumina 610 quad	Sample QC: Sample callrate >97.5%, no excess autosomal heterozygosity (<0.336), no sex-mismatch. SNPs QC: SNP callrate >98% MAF >0.01, HWE> 1x10E-6	MAF ≥ 0,001 ; imputation quality ≥0,3	HRC r1
RS-II	illumina 550 duo	As RS-I	MAF ≥ 0,001 ; imputation quality ≥0,3	HRC r1
RS-III	illumina 610 quad	As RS-I	MAF ≥ 0,001 ; imputation quality ≥0,3	HRC r1
TEST-BATS	Human610-Quadv1_B(610K)	mean(GenCall score)>0.7; MAF > 1%; call rate >0.95; HWE> 1x10E-6	MAF ≥ 0.001 ; imputation quality ≥0.3	HRC r1
TwinsUK	Illumina 317K Duo and HumanHap 610K-Quad	MAF > 0.001; SNPcallrate >0.95; HWE> 1x10E-6	NA	HRC r1.1
VIKING	OmniExpressExome-8v1-2_A	MAF > 0.01; SNPcallrate >0.98; HWE> 1x10E-6	Monomorphic, duplicate and triallelic removed ; imputation quality-INFO ≥0.4	HRC r1

Supplementary Table 8 Baseline characteristics: ancestry and study specific covariates.

Study	Ancestry	Study specific covariates used in analysis
CROATIA-Korcula	European,Croatian	Family structure
CROATIA-Vis	European, Croatian	Family structure
EPIC-Norfolk Eye Study	European, English	
ERF	European, Dutch	Family structure
GHS1	European, German	
GHS2	European, German	
ORCADES	European, Orcadian	Family structure
RS-I	European, Dutch	
RS-II	European, Dutch	
RS-III	European, Dutch	
TEST-BATS	European, Australia	Measurement Technique of IOP: Tonopen and GAT
VIKING	European, Shetlandic	Family structure

Supplementary Table 9 SNP genotyping and imputation details of the POAG case-control studies

MAF = minor allele frequency; HWE = Hardy-Weinberg equilibrium

Study	Genotype platform	Pre imputation QC thresholds	Post imputation QC thresholds	HRC version
NEIGHBOR/MEEI	Illumina 660W_Quad_v1 array	MAF > 0.01; SNPcallrate >0.95	MAF > 0.01; imputation quality > 0.7	HRC r1
Southampton	Affymetrix SNP6.0	MAF > 0.05; SNPcallrate > 0.90; HWE > 1x10E-10 (cases)	MAF > 0.01 ; imputation quality ≥0,3	HRC r1.1
UK Biobank	UK BiLEVE Affymetrix Axiom	SNPcallrate >0.95; HWE> 1x10E-6	MAF > 0.005; imputation quality > 0.4	HRC

Supplementary Note

1.1 study descriptives European population-based studies

CROATIA-Korcula and CROATIA-Vis. The CROATIA-Vis study includes unselected adult participants who were recruited in a population-based study during 2003 and 2004 in the villages of Vis and Komiza on the Dalmatian island of Vis. All subjects visited the clinical research center in the region where they were examined in person and where fasting blood was drawn. Biochemical and physiological measurements were performed, detailed genealogies reconstructed, questionnaire of lifestyle and environmental exposures collected, and blood samples stored for further analyses. CROATIA-Korcula participants were recruited in the same manner from the Dalmatian island of Korcula in 2007. All studies received appropriate ethical approval, and all participants gave informed consent.

EPIC-Norfolk Eye Study. The European Prospective Investigation into Cancer (EPIC) study is a pan-European prospective cohort study designed to investigate the etiology of major chronic diseases [3]. EPIC-Norfolk , one of the UK arms of EPIC, recruited and examined 25,639 participants between 1993 and 1997 for the baseline examination[4]. Recruitment was via general practices in the city of Norwich and the surrounding small towns and rural areas, and methods have been described in detail previously[5]. Since virtually all residents in the UK are registered with a general practitioner through the National Health Service, general practice lists serve as population registers. Ophthalmic assessment formed part of the third health examination and this has been termed the EPIC-Norfolk Eye Study [6]. In total, 8,623 participants were seen for the Eye Study, between 2004 and 2011. The EPIC-Norfolk Eye Study was carried out following the principles of the Declaration of Helsinki and the Research Governance Framework for Health and Social Care. The study was approved by the Norfolk Local Research Ethics Committee (05/Q0101/191) and East Norfolk & Waveney NHS Research Governance Committee (2005EC07L). All participants gave written, informed consent.

Erasmus Rucphen Family (ERF) Study. The ERF study has been described in detail previously[7]. A total of approximately 3,000 participants descend from 22 couples who lived in the Rucphen region in The Netherlands in the 19th century. The 2,755 individuals with genotype data and lipid measurements were included in the current analysis.

Gutenberg Health Study I and II (GHS I and GHS II). The GHS is a population-based, prospective, observational cohort study in the Rhine-Main Region in midwestern Germany with a total of 15,010 participants and follow-up after five years. The study sample is recruited from subjects aged between 35 and 74 years at the time of the exam. The sample was drawn randomly from local governmental registry offices and stratified by gender, residence (urban and rural) and decade of age. Exclusion criteria were insufficient knowledge of the German language to understand explanations and instructions, and physical or psychic inability to participate in the examinations in the study center. The study was approved by the Medical Ethics Committee of the University Medical Center Mainz and by the local and federal data safety commissioners. According to the tenets of the Declaration of Helsinki, written informed consent was obtained from all participants prior to entering the study.

Orkney Complex Disease studies (ORCADES). The ORCADES is a family-based, cross-sectional community study of the genetics of complex traits, based in the Orkney Isles in Scotland[8]. All participants gave written informed consent, in accord with the tenets of the declaration of Helsinki, and the study was approved by Research Ethics Committees in Orkney and Aberdeen (North of Scotland REC).

Rotterdam Study cohort I (RS-I). The Rotterdam Study is an ongoing prospective population-based cohort study, focused on chronic disabling conditions of the elderly. The study comprises an outbred ethnically homogenous population of Dutch Caucasian origin. The rationale of the study has been described in detail elsewhere[9]. In summary, 7,983 men and women aged 55 years or older, living in Ommoord, a suburb of Rotterdam, the Netherlands, were invited to participate in the first phase. Fasting blood samples were taken during the participant's third visit to the research center.

Rotterdam Study cohort II (RS-II). The Rotterdam Study cohort II prospective population-based cohort study comprises 3,011 residents aged 55 years and older from the same district of Rotterdam. The rationale and study designs of this cohort is similar to that of the RS-I[9]. The baseline measurements, including the fasting HDL measurements, took place during the first visit.

Rotterdam Study cohort III (RS-III). The Rotterdam Study cohort III prospective population-based cohort study

comprised 3,932 residents aged 45 years and older from the same district of Rotterdam. The rationale and study designs of this cohort is similar to that of the RS-I[9]. The baseline measurements, including the fasting HDL measurements, took place during the first visit.

Twins Eye Study in Tasmania (TEST), Brisbane Adolescent Twin Study (BATS).
The Australian Twin Eye Study comprises participants examined as part of TEST or BATS. The studies were approved by the human ethics committees of the University of Tasmania, Royal Victorian Eye and Ear Hospital, and Queensland Institute of Medical Research.

TwinsUK. The TwinsUK adult twin registry based at St. Thomas' Hospital in London is a volunteer cohort of over 10,000 twins from the general population [10]. Twins largely volunteered unaware of the eye studies, gave fully informed consent under a protocol reviewed by the St. Thomas' Hospital Local Research Ethics Committee. Out of the original 1,951 subjects for whom phenotype and genotype information was available, 1,922 subjects were included in the study; 29 subjects were excluded after failing quality control.

VIKING. The Viking Health Study - Shetland (VIKING) is a family-based, cross-sectional study that seeks to identify genetic factors influencing cardiovascular and other disease risk in the population isolate of the Shetland Isles in northern Scotland. Genetic diversity in this population is decreased compared to Mainland Scotland, consistent with the high levels of endogamy historically. 2105 participants were recruited between 2013 and 2015, each having at least three grandparents from Shetland. All participants gave written informed consent and the study was approved by the South East Scotland Research Ethics Committee.

1.2 study descriptives Asian population-based studies

Beijing Eye Study (BES). The BES is a population-based cohort of Han Chinese in the rural region and in the urban region of Beijing in North China [11,12]. The Medical Ethics Committee of the Beijing Tongren Hospital approved the study protocol and all participants gave informed consent, according to the Declaration of Helsinki. At baseline (2001), 4439 individuals out of 5324 eligible individuals aged 40 years or older participated (response rate: 83.4%). In the years 2006 and 2011, the study was repeated by re-inviting all participants from the survey from 2001 to be re-examined. Out of the 4439 subjects examined in 2001, 3251 (73.2%) subjects returned for the follow-up examination in 2006, and 2695 (60.7%) subjects returned for the follow-up examination in 2011.

Singapore Malay Eye Study (SIMES), Singapore Indian Eye Study (SINDI), and Singapore Chinese Eye Study (SCES)
The three population-based studies were conducted in Singapore: the Singapore Malay Eye Study from 2004 to 2006, the Singapore Indian Eye Study from 2007 to 2009, and the Singapore Chinese Eye Study from 2009 to 2011. The detailed methodology of these three study has been published in previous reports [13,14].In brief, an age-stratified (by 10-year age groups) random sampling in each ethnic group was used to select ethnic Malays, Indians, and Chinese 40 to 80 years of age living across the southwestern part of Singapore during each stipulated study period. The number of selected subjects was 4,168 Malays, 4,497 Indians and 4,606 Chinese. Of these, 3280 Malay persons (response rate 78.7%), 3,400 Indian persons (75.6%) and 3,353 Chinese persons (72.8%) participated in the study. The overall response rate for SEED was 75.6%. This study was approved by the Singapore Eye Research Institute Institutional Review Board and the conduct of the study adhered to the Declaration of Helsinki. All participants gave informed consent. All subjects recruited in the SEED study underwent a standardized interview, and ocular examination at the Singapore Eye Research Institute. An interviewer-administered questionnaire was used to collect demographic data.

1.3 study descriptives & methods European POAG case-controls studies

NEIGHBOR and MEEI
All cases and controls met the clinical criteria used previously by the NEIGHBOR and GLAUGEN studies previously described (Wiggs et al., 2012; Wiggs et al., 2013; Bailey et al., 2016). This study Subjects were enrolled using a protocol was approved by the Massachusetts Eye and Ear Infirmary institutional review board and all subjects signed consent forms approved by the local IRB prior to enrolling in the study. Briefly, POAG cases were defined as individuals for whom reliable visual field (VF) tests showed characteristic VF defects consistent with glaucomatous optic neuropathy. Individuals were classified as affected if the VF defects were reproduced on a subsequent test or if a single qualifying VF was accompanied by a cup-disc ratio (CDR) of 0.7 or more in at least one eye. The majority of cases (over 90%) met this definition, including 96% of the NEIGHBOR cases (Wiggs et al., J of Glaucoma, 2013); and

all of the Mass Eye and Ear, NHS, HPFS, and WGHS cases. A small percentage (less than 10%) of the NEIGHBOR, cases were defined by cup-to-disc ratio only because visual field data was not available, in some cases because of advanced disease (poor visual acuity) or other medical condition. The CDR definition was > 0.7 in both eyes or CDR asymmetry between the two eye of 0.2 (Supplementary Table 2). Patients with signs of secondary causes for elevated IOP such as exfoliation syndrome or pigment dispersion syndrome or critically narrow filtration structures were excluded. Elevation of IOP was not a criterion for inclusion of cases or controls Controls had IOP < 21 mmHg, as measured in a clinical setting, CDR of less than 0.6 and did not have a family history of glaucoma.
Imputed genotypes (Haplotype Reference Consortium) for 2,606 cases and 2,606 controls from 2 independent datasets (NEIGHBOR and MEEI) were used for the replication effort. Quality-control was performed for each data set as described in Bailey et al., 2016 (Supplemental Note).

Southampton. Primary open-angle (POAG) and normal tension glaucoma patients were recruited from the Southampton University Hospital Trust Eye Clinic and satellite regional glaucoma clinics. Ethical approval for the collection of patient information and blood samples was provided by the Southampton and South West Hampshire Local Research Ethics Committee (05/Q1702/8) and Cohort Recruitment commenced in August 2005. Each patient was examined by an experienced glaucoma specialist. Diagnoses were made on the basis of characteristic visual field loss/glaucomatous optic disc damage/increased IOP. Patients presenting with narrow-angle, developmental or secondary glaucoma or any other known abnormalities of the anterior segment were excluded. Patients with unambiguous glaucoma, but normal tension were included in sample collection later. Furthermore, to select for patients with typical POAG or normal-tension glaucoma (NTG), only patients diagnosed over the age of 40 years were included. Both conditions are rare before this age. DNA was extracted according to the standard methods, dissolved in TE buffer, and stored at -20°C. Primary open angle glaucoma patients (n=400) were genotyped on the Affymetrix SNP 6.0 array, all data were exported on the forward strand. These data were combined with the Affymetrix SNP 6.0 data publically available for the WTCCC2 controls. The Genome-wide association data previously described[47] was further filtered to ensure removal of individuals with more than 5% missing genotypes, SNPs with more than 3% missing samples, SNPs with a minor allele frequency of <1% and a Hardy-Weinberg p-value < 1x10-6. SNPs were all on the forward strand and locations were lifted over from hg18 to hg19 using the UCSC liftover tool. SNPs with complementary alleles were also excluded (A/T and G/C).
The data used for imputation included 384 cases and 3389 controls, and 533,774 SNPs. Pre-phasing was carried out using Shapeit (v2, r790) and imputation was carried out using Impute2 (v2.3.1), using 1000 Genomes Phase I integrated haplotypes (produced using SHAPEIT2), b37 Dec 2013, downloaded from the impute2 website. Imputation was carried out over the genome in 5Mb chunks as per the best practices on the impute2 website. The imputed data was then converted to plink format using GTOOL. Case-control analysis was carried out using logistic regressions for the selected replication SNPs and Indels with sex as a covariate, using PLINK (v1.90b3b 64-bit (15 Jan 2015)).

UK biobank

Self-reported glaucoma status was ascertained by participant selection of 'glaucoma' from a list of eye disorders in response to the question "Has a doctor told you that you have any of the following problems with your eyes?". Cases were defined as those reporting "glaucoma." The control group included the non-cases and excluded subjects who reported "Prefer not to answer" or "Do not know." Genotyping was done using two arrays. The first array was the UK BiLEVE [15]. The second array is what was the Affymetrix Axiom® platform with a custom-designed array described in the UK Biobank Axiom® Array Content Summary (http://www.ukbiobank.ac.uk/wp-content/uploads/2014/04/UK-Biobank-Axiom-Array-Content-Summary- 2014.pdf). Phasing on the autosomes was carried out using a modified version of the SHAPEIT22 program modified to allow for very large sample sizes [16]. A total of 806,466 directly genotyped DNA sequence variants were available after variant quality control. The UK Biobank team then performed imputation on Haplotype Reference Consortium (HRC) reference panel; phasing was performed using SHAPEIT3 and imputation was carried out via the IMPUTE4 program [17]. The variant-level quality control exclusion metrics applied to imputed data for GWAS included the following: call rate < 95%, Hardy–Weinberg equilibrium $P < 1 \times 10^{-6}$, posterior call probability < 0.9, imputation quality < 0.4, and MAF < 0.005. Sex chromosome and mitochondrial genetic data were excluded from this analysis. In total, 9,061,845 imputed DNA sequence variants were included in our analysis. For sample quality control, we removed individuals with relatedness corresponding to third-degree relatives or closer, and an additional samples with an excess of missing genotype calls or more heterozygosity than expected were excluded.

Acknowledgements

Beijing Eye Study (JJES)
Beijing Eye Study was supported by National Natural Science Foundation of China (grant 81170890).

CROATIA studies
The CROATIA studies were funded by grants from the Medical Research Council (UK), from the Republic of Croatia Ministry of Science, Education and Sports (108-1080315-0302; 216-1080315-0302) and the Croatian Science Foundation (8875); and the CROATIA-Korčula genotyping was funded by the European Union framework program 6 project EUROSPAN (LSHGCT2006018947). The CROATIA studies acknowledges Dr. Biljana Andrijević Derk, Valentina Lacmanović Lončar, Krešimir Mandić, Antonija Mandić, Ivan Škegro, Jasna Pavičić Astaloš, Ivana Merc, Miljenka Martinović, Petra Kralj, Tamara Knežević and Katja Barać-Juretić as well as the recruitment team from the Croatian Centre for Global Health, University of Split and the Institute of Anthropological Research in Zagreb for the ophthalmological data collection; Peter Lichner and the Helmholtz Zentrum Munchen (Munich, Germany), AROS Applied Biotechnology, Aarhus, Denmark and the Edinburgh Clinical Research facility, University of Edinburgh, for SNP array genotyping; . Genetic analyses were supported by the MRC HGU "QTL in Health and Disease" core programme.

ORCADES
ORCADES The Orkney Complex Disease Study (ORCADES) was supported by the Chief Scientist Office of the Scottish Government (CZB/4/276, CZB/4/710), a Royal Society URF to J.F.W., the MRC Human Genetics Unit quinquennial programme "QTL in Health and Disease", Arthritis Research UK and the European Union framework program 6 EUROSPAN project (contract no. LSHG-CT-2006-018947). DNA extractions were performed at the Edinburgh Clinical Research Facility, University of Edinburgh. We would like to acknowledge the invaluable contributions of the research nurses in Orkney, the administrative team in Edinburgh and the people of Orkney.. Genetic analyses were supported by the MRC HGU "QTL in Health and Disease" core programme.

EPIC-Norfolk Eye study
EPIC-Norfolk infrastructure and core functions are supported by grants from the Medical Research Council (G1000143) and Cancer Research UK (C864/A14136). The clinic for the third health examination was funded by Research into Ageing (262). Genotyping was funded by the Medical research Council (MC_PC_13048). We thank all staff from the MRC Epidemiology laboratory team for the preparation and quality control of DNA samples. Mr Khawaja is supported by a Moorfields Eye Charity Fellowship. Miss Chan is a joint Medical Research Council/Royal College of Ophthalmologists Research Fellow, and received additional support from the International Glaucoma Association. Professor Foster has received additional support from the Richard Desmond Charitable Trust (via Fight for Sight) and the Department for Health through the award made by the National Institute for Health Research to Moorfields Eye Hospital and the UCL Institute of Ophthalmology for a specialist Biomedical Research Centre for Ophthalmology.

Erasmus Rucphen Family (ERF) Study and Rotterdam Study
Erasmus Rucphen family study: The ERF study as a part of EUROSPAN (European Special Populations Research Network) was supported by European Commission FP6 STRP grant number 018947 (LSHG-CT-2006-01947) and also received funding from the European Community's Seventh Framework Programme (FP7/2007-2013)/grant agreement HEALTH- F4-2007-201413 by the European Commission under the program 'Quality of Life and Management of the Living Resources' of the 5th Framework Programme (number QLG2-CT- 2002-012.
The Rotterdam Study is funded by Erasmus Medical Center and Erasmus University, Rotterdam, Netherlands Organization for the Health Research and Development (ZonMw), the Research Institute for Diseases in the Elderly (RIDE), the Ministry of Education, Culture and Science, the Ministry for Health, Welfare and Sports, the European Commission (DG XII), and the Municipality of Rotterdam. The generation and management of GWAS genotype data for the Rotterdam Study (RS-I, RS-II, RS-III) was executed by the Human Genotyping Facility of the Genetic Laboratory of the Department of Internal Medicine, Erasmus MC, Rotterdam, The Netherlands. The GWAS datasets are supported by the Netherlands Organization of Scientific Research NWO Investments (nr. 175.010.2005.011, 911-03-012), the Genetic Laboratory of the Department of Internal Medicine, Erasmus MC, the Research Institute for Diseases in the Elderly (014-93-015; RIDE2), the Netherlands Genomics Initiative (NGI)/Netherlands Organization for Scientific Research (NWO) Netherlands Consortium for Healthy Aging (NCHA), project nr. 050-060-810. This study makes use of an extended dataset of RS-II and RS-III samples based on Illumina Omni 2.5 and 5.0 GWAS genotype data. This dataset was funded by the Genetic Laboratory of the Department of Internal Medicine, the Department of Forensic Molecular Biology, and the Department of Dermatology, Erasmus MC, Rotterdam, The Netherlands. We

thank Pascal Arp, Mila Jhamai, Marijn Verkerk, Lizbeth Herrera and Marjolein Peters, MSc, and Carolina Medina-Gomez, PhD, for their help in creating the GWAS database. A.I. Iglesias was supported by the National Institute of Health (NIH), National Eye Institute (NEI), (1 R01 EY024233-03).

National Eye Institute (NEI) Glaucoma Human Genetics Collaboration (NEIGHBOR)
Genotyping services for the NEIGHBOR study were provided by the Center for Inherited Disease Research (CIDR) and were supported by the National Eye Institute through grant HG005259-01 (JL Wiggs). Additionally, CIDR is funded through a federal contract from the National Institutes of Health to The Johns Hopkins University, contract number HHSN268200782096C. Collecting and processing samples for the NEIGHBOR dataset was supported by the National Eye Institute through ARRA grants 3R01EY015872-05S1 (JL Wiggs) and 3R01EY019126-02S1 (MA Hauser). The NEIGHBOR and MEEI datasets used in this study are part of the NEIGHBORHOOD consortium supported by NIH/NEI R01EY022305.

Gutenberg Health Study (GHS I, GHS II)
The Gutenberg Health Study was funded through the government of Rhineland-Palatine ("Stiftung Rheinland Pfalz für Innovation" (AZ961386261733); the research programs "Wissen schafft Zukunft" 67 and "Schwerpunkt Vaskuläre Prävention" of the Johannes Gutenberg-University of Mainz; Boehringer Ingelheim, Ingelheim, Germany; PHILIPS Medical Systems, Hamburg, Germany; National Genome Network ''NGFNplus'' by the Federal Ministry of Education and Research, Germany (A301GS0833).

Singapore (SCES, SIMES, SINDI)
The Singapore Chinese Eye Study (SCES), Singapore Malay Eye Study (SiMES) and Singapore Indian Eye Study (SINDI) were supported by the National Medical Research Council (NMRC), Singapore (NMRC/0796/2003, NMRC/1249/2010, NMRC/CIRG/1371/2013, NMRC/CIRG/1417/2015 and NMRC/CSA-SI/0012/2017); and Biomedical Research Council (BMRC), Singapore (BMRC 08/1/35/19/550). Dr. Ching-Yu Cheng is supported by an award from NMRC (CSA-SI/0012/2017).

Twins Eye Study in Tasmania (TEST) and Brisbane Adolescent Twin Study (BATS)
TEST and BATS (Australian Twins) were supported by an Australian National Health and Medical Research Council (NHMRC) Enabling Grant (2004-2009, 350415, 2005-2007); Clifford Craig Medical Research Trust; Ophthalmic Research Institute of Australia; American Health Assistance Foundation; Peggy and Leslie Cranbourne Foundation; Foundation for Children; Jack Brockhoff Foundation; National Institutes of Health/National Eye Institute (RO1EY01824601 (2007-2010)); Pfizer Australia Senior Research Fellowship (to D.A.M.); and Australian NHMRC Career Development Award (to S.M.). Genotyping was funded by an NHMRC Medical Genomics Grant; US National Institutes of Health/National Eye Institute (1RO1EY018246), Australian sample imputation analyses were carried out on the Genetic Cluster Computer which is financially supported by the Netherlands Scientific Organization (NWO48005003). Australian Twins thanks Nicholas Martin, Scott Gordon, Dale Nyholt, Sarah Medland, Brian McEvoy, Margaret Wright, Anjali Henders, Megan Campbell for ascertaining and processing genotyping data; Jane MacKinnon, Shayne Brown, Lisa Kearns, Jonathan Ruddle, 68 Sandra Staffieri, Olivia Bigault, Colleen Wilkinson, Byoung Sung Chu, Robert Macmillan, Johan Poulsen, Yaling Ma, Julie Barbour for assisting with clinical examinations; and Dr Camilla Day and staff at the Center for Inherited Disease Research.

TwinsUK
TwinsUK received funding from the Wellcome Trust; the European Union MyEuropia Marie Curie Research Training Network; Guide Dogs for the Blind Association; the European Community's FP7 (HEALTHF22008201865GEFOS); ENGAGE (HEALTHF42007201413);
the FP-5 GenomEUtwin Project (QLG2CT200201254); US National Institutes of Health/National Eye Institute (1RO1EY018246); NIH Center for Inherited Disease Research; the National Institute for Health Research comprehensive Biomedical Research Centre award to Guy's and St. Thomas' National Health Service Foundation Trust partnering with King's College London. A.N. received funding from Fight for Sight and The Worshipful Company of Spectacle Makers. P.G.H. is the recipient of a Fight for Sight ECI award. C.J.H. is an NIHR Senior Research fellow. We acknowledge the contribution of Drs Toby Andrew, Margarida Lopes, Samantha Fahy and Diana Kozareva.

VIKING
The Viking Health Study – Shetland (VIKING) was supported by the MRC Human Genetics Unit quinquennial programme grant "QTL in Health and Disease". DNA extractions and genotyping were performed at the Edinburgh Clinical Research Facility, University of Edinburgh. We would like to acknowledge the invaluable contributions of the

research nurses in Shetland, the administrative team in Edinburgh and the people of Shetland.

References

1. Springelkamp, H. *et al.* Meta-analysis of Genome-Wide Association Studies Identifies Novel Loci Associated With Optic Disc Morphology. *Genet Epidemiol* **39**, 207-16 (2015).
2. Iglesias, A.I. *et al.* Cross-ancestry genome-wide association analysis of corneal thickness strengthens link between complex and Mendelian eye diseases. *Nat Commun* **9**, 1864 (2018).
3. Riboli, E. & Kaaks, R. The EPIC Project: rationale and study design. European Prospective Investigation into Cancer and Nutrition. *Int J Epidemiol* **26 Suppl 1**, S6-14 (1997).
4. Day, N. *et al.* EPIC-Norfolk: study design and characteristics of the cohort. European Prospective Investigation of Cancer. *Br J Cancer* **80 Suppl 1**, 95-103 (1999).
5. Hayat, S.A. *et al.* Cohort profile: A prospective cohort study of objective physical and cognitive capability and visual health in an ageing population of men and women in Norfolk (EPIC-Norfolk 3). *Int J Epidemiol* **43**, 1063-72 (2014).
6. Khawaja, A.P. *et al.* The EPIC-Norfolk Eye Study: rationale, methods and a cross-sectional analysis of visual impairment in a population-based cohort. *BMJ Open* **3**(2013).
7. Pardo, L.M., MacKay, I., Oostra, B., van Duijn, C.M. & Aulchenko, Y.S. The effect of genetic drift in a young genetically isolated population. *Ann Hum Genet* **69**, 288-95 (2005).
8. McQuillan, R. *et al.* Runs of homozygosity in European populations. *Am J Hum Genet* **83**, 359-72 (2008).
9. Hofman, A. *et al.* The Rotterdam Study: 2016 objectives and design update. *Eur J Epidemiol* **30**, 661-708 (2015).
10. Spector, T.D. & Williams, F.M. The UK Adult Twin Registry (TwinsUK). *Twin Res Hum Genet* **9**, 899-906 (2006).
11. Xu, L. *et al.* Visual acuity in northern China in an urban and rural population: the Beijing Eye Study. *Br J Ophthalmol* **89**, 1089-93 (2005).
12. Xu, L., Zhang, H., Wang, Y.X. & Jonas, J.B. Central corneal thickness and glaucoma in adult Chinese: the Beijing Eye Study. *J Glaucoma* **17**, 647-53 (2008).
13. Lavanya, R. *et al.* Methodology of the Singapore Indian Chinese Cohort (SICC) eye study: quantifying ethnic variations in the epidemiology of eye diseases in Asians. *Ophthalmic Epidemiol* **16**, 325-36 (2009).
14. Foong, A.W. *et al.* Rationale and methodology for a population-based study of eye diseases in Malay people: The Singapore Malay eye study (SiMES). *Ophthalmic Epidemiol* **14**, 25-35 (2007).
15. Wain, L.V. *et al.* Novel insights into the genetics of smoking behaviour, lung function, and chronic obstructive pulmonary disease (UK BiLEVE): a genetic association study in UK Biobank. *Lancet Respir Med* **3**, 769-81 (2015).
16. O'Connell, J. *et al.* Haplotype estimation for biobank-scale data sets. *Nat Genet* **48**, 817-20 (2016).
17. Howie, B.N., Donnelly, P. & Marchini, J. A flexible and accurate genotype imputation method for the next generation of genome-wide association studies. *PLoS Genet* **5**, e1000529 (2009).
