# Supplementary material for: Multi-trait genome-wide association study identifies new loci associated with optic disc parameters
Source: Commun Biol. 2019 Nov 27;2:435. doi: 10.1038/s42003-019-0634-9 (PMC6881308; doi:10.1038/s42003-019-0634-9)
Supplement: Supplementary file 2 — Description of Additional Supplementary Files [file 42003_2019_634_MOESM2_ESM.docx]

descriptions of additional supplementary files

***Multi-trait GWAS identifies new loci associated with optic disc parameters***

Supplementary Data

Supplementary Data 1 *Summary of replicated 1000Genomes identified loci*
Supplementary Data 2 *Replication results of 1000G loci in current HRC imputed meta-analyses*
Supplementary Data 3 *Test statistic inflation and SNP-based heritability for the five meta-analysis*
Supplementary Data 4 *results of GWAS meta-analysis and replication in Asian sample*Supplementary Data 5 *Table 1 with complete per cohort statistics*Supplementary Data 6 *Results from multi-trait analysis for CA,DA,VCDR by CPASSOC*
Supplementary Data 7 *Validation of CPASSOC CA,DA,VCDR results by MTAG*
Supplementary Data 8 *Results from multi-trait analysis for IOP,CCT by CPASSOC*
Supplementary Data 9 *Validation of CPASSOC IOP,CCT results by MTAG*
Supplementary Data 10 *Bioinformatical lookup*
Supplementary Data 11 *Gene prioritization methods and assessment of expression in eye tissues*
Supplementary Data 12 *Pathway analysis for ONH traits, IOP and CCT*
Supplementary Data 13 *results per POAG study for rs1028727 and rs11158547*
Supplementary Data 14 *Validation in meta-analysis of 3 POAG studies*
