# Supplementary material for: Multi-trait genome-wide association study identifies new loci associated with optic disc parameters
Source: Commun Biol. 2019 Nov 27;2:435. doi: 10.1038/s42003-019-0634-9 (PMC6881308; doi:10.1038/s42003-019-0634-9)
Supplement: Supplementary file 4 — Reporting Summary [file 42003_2019_634_MOESM4_ESM.pdf]

nature research

Corresponding author(s): Cornelia van Duijn

Last updated by author(s): Aug 31, 2019

Reporting Summary

Nature Research wishes to improve the reproducibility of the work that we publish. This form provides structure for consistency and transparency in reporting. For further information on Nature Research policies, see Authors & Referees and the Editorial Policy Checklist.

Statistics

For all statistical analyses, confirm that the following items are present in the figure legend, table legend, main text, or Methods section.

n/a	Confirmed	
☐	☒	The exact sample size (*n*) for each experimental group/condition, given as a discrete number and unit of measurement
☐	☒	A statement on whether measurements were taken from distinct samples or whether the same sample was measured repeatedly
☐	☒	The statistical test(s) used AND whether they are one- or two-sided<br>*Only common tests should be described solely by name; describe more complex techniques in the Methods section.*
☐	☒	A description of all covariates tested
☐	☒	A description of any assumptions or corrections, such as tests of normality and adjustment for multiple comparisons
☐	☒	A full description of the statistical parameters including central tendency (e.g. means) or other basic estimates (e.g. regression coefficient) AND variation (e.g. standard deviation) or associated estimates of uncertainty (e.g. confidence intervals)
☐	☒	For null hypothesis testing, the test statistic (e.g. *F*, *t*, *r*) with confidence intervals, effect sizes, degrees of freedom and *P* value noted<br>*Give P values as exact values whenever suitable.*
☒	☐	For Bayesian analysis, information on the choice of priors and Markov chain Monte Carlo settings
☒	☐	For hierarchical and complex designs, identification of the appropriate level for tests and full reporting of outcomes
☐	☒	Estimates of effect sizes (e.g. Cohen's *d*, Pearson's *r*), indicating how they were calculated

Our web collection on statistics for biologists contains articles on many of the points above.

Software and code

Policy information about availability of computer code

Data collection	No software was used for the data collection of this study.
Data analysis	METAL (https://genome.sph.umich.edu/wiki/METAL), EasyQC (www.genepi-regensburg.de/easyqc), GCTA (http://cnsgenomics.com/software/gcta/), FUMA (https://fuma.ctglab.nl/),LDSC (https://github.com/bulik/ldsc), CPASSOC (http://hal.case.edu/zhu-web/), MTAG (https://github.com/omeed-maghzian/mtag)

For manuscripts utilizing custom algorithms or software that are central to the research but not yet described in published literature, software must be made available to editors/reviewers. We strongly encourage code deposition in a community repository (e.g. GitHub). See the Nature Research guidelines for submitting code & software for further information.

Data

Policy information about availability of data

All manuscripts must include a data availability statement. This statement should provide the following information, where applicable:

- Accession codes, unique identifiers, or web links for publicly available datasets
- A list of figures that have associated raw data
- A description of any restrictions on data availability

The genome-wide summary statistics that support the findings of this study will be made available via the NHGRI-EBI GWAS Catalog website (https://www.ebi.ac.uk/gwas/downloads/summarystatistics) upon publication.

Field-specific reporting

Please select the one below that is the best fit for your research. If you are not sure, read the appropriate sections before making your selection.

☒ Life sciences ☐ Behavioural & social sciences ☐ Ecological, evolutionary & environmental sciences

For a reference copy of the document with all sections, see nature.com/documents/nr-reporting-summary-flat.pdf

Life sciences study design

All studies must disclose on these points even when the disclosure is negative.

Sample size	No sample size calculation was performed. We aimed to include all cohorts within the International Glaucoma Genetics Consortium with available data for cup area, disc area, vertical cup disc ratio, intraocular pressure and central corneal thickness
Data exclusions	Quality and control procedures are described in the methods
Replication	Methods of replication are melticously described in the methods
Randomization	This is not relevant to our study since no randomization was performed.
Blinding	This is not relevant to our study since no blinding was performed.

Reporting for specific materials, systems and methods

We require information from authors about some types of materials, experimental systems and methods used in many studies. Here, indicate whether each material, system or method listed is relevant to your study. If you are not sure if a list item applies to your research, read the appropriate section before selecting a response.

Materials & experimental systems

n/a	Involved in the study	
☒	☐	Antibodies
☒	☐	Eukaryotic cell lines
☒	☐	Palaeontology
☒	☐	Animals and other organisms
☐	☒	Human research participants
☒	☐	Clinical data

Methods

n/a	Involved in the study	
☒	☐	ChIP-seq
☒	☐	Flow cytometry
☒	☐	MRI-based neuroimaging

Human research participants

Policy information about studies involving human research participants

Population characteristics	These information is elaborately described in the Supplementary information appending the manuscript
Recruitment	The recruitment of the studies differed; for more information please refer to the Supplementary note
Ethics oversight	Norfolk Local Research Ethics Committee (05/Q0101/191) and East Norfolk & Waveney NHS Research Governance Committee (2005EC07L), the Medical Ethics Committee of the University Medical Center Mainz, Research Ethics Committees in Orkney and Aberdeen (North of Scotland REC), human ethics committees of the University of Tasmania, Royal Victorian Eye and Ear Hospital, and Queensland Institute of Medical Research, Erasmus MC, St. Thomas' Hospital Local Research Ethics Committee, South East Scotland Research Ethics Committee, Medical Ethics Committee of the Beijing Tongren Hospital, Singapore Eye Research Institute Institutional Review Board, the Massachusetts Eye and Ear Infirmary institutional review board, the Southampton and South West Hampshire Local Research Ethics Committee (05/Q1702/8), North-West Research Ethics Committee (ref 06/MRE08/65),

Note that full information on the approval of the study protocol must also be provided in the manuscript.
